# Batch Heterogeneous Catalytic Selective Hydrogenation of Vegetable Oils Over Lindlar Catalyst: Kinetic Modeling Supported by Reaction Mechanisms

**DOI:** 10.1002/open.202500369

**Published:** 2025-09-24

**Authors:** Enza Pellegrino, Katia Gallucci, Nicoletta Cancrini, Andrea Di Giuliano

**Affiliations:** ^1^ Department of Industrial and Information Engineering and Economics Università degli Studi dell’Aquila Piazzale E. Pontieri 1 loc. Monteluco di Roio 67100 L’Aquila (AQ) Italy

**Keywords:** catalysis, Hougen‐Watson, reaction mechanism, selective hydrogenation, vegetable oil

## Abstract

Heterogeneous catalytic selective hydrogenation (HCSH) of vegetable oils is a reactive pathway to maximize the fraction of monounsaturated oleic acid, a component of industrial interest, thanks to its stability and low‐temperature properties. Kinetics of HCSH is usually interpreted by pseudo‐first order laws, as previously done by this research group with data from batch tests on canola oil (Lindlar catalyst, 120–180 °C, 0.4–1.2 MPa): the pseudo‐first order kinetics could interpret the observed phenomenon only by variable selectivities (i.e., ratios of kinetic constants). The present work proposes a refined modeling by Hougen–Watson approach. Detailed reaction mechanisms were hypothesized, challenging the following alternatives: (i) H_2_ adsorption on the catalyst occured (Hinshelwood–Langmuir) or not (Eley–Rideal), (ii) H_2_ adsorption was molecular or dissociative, (iii) H_2_ adsorption was competitive or not with fatty acids adsorption, and (iv) reaction intermediates were formed or not. Six reaction mechanisms were developed with related kinetic rate laws for HCSH. Their kinetic parameters were regressed for the abovementioned experimental data by a purposely developed MATLAB script. The best mechanism was chosen based on the highest explained variance ($R^{2}$) and the lowest number of parameters. Chemical–physical meaningfulness of regressed parameters for the best model was checked by comparison with the literature.

## Graphical abstract

Heterogeneous catalytic selective hydrogenation of vegetable oils was modeled by Hougen–Watson approach to obtain kinetic rate laws. Parameters were obtained by nonlinear regressions on batch experimental data, and the best mechanism to interpret those experimental data was chosen on a statistical basis (higher coefficient of determination R^2^) and by Occam's razor. Chemical–physical meaningfulness of mechanisms and parameters was discussed comparatively with the literature.

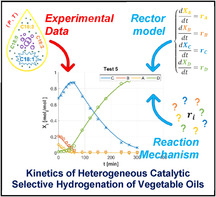

“*Truth…is much too complicated to allow anything but approximations*”

John von Neumann (1903–1957)

Hungarian‐born American mathematician and computer pioneer.

## 1 Introduction

Vegetable oils (VO) mainly consist of 18‐carbon‐atoms fatty acids with an ‘i’ number of unsaturations (i.e., double bonds), referred to as C18:i in the following. The C18:i in VO are esterified into triglycerides or free. They are of interest in the field of Green Chemistry and Green Engineering as they can be transformed into sustainable added‐value molecules, e.g., building blocks for bio‐plastics or biolubricants.^[^ ^1^ ^,^ ^2^ ^]^

Chemical reaction pathways can be used to modulate the properties of VO.^[^ ^2^ ^]^ For instance, the monounsaturated oleic acid (C18:1) is of industrial interest due to its higher chemical stability and lower solidification point in comparison to other fatty acids.^[^ ^1^ ^]^ The heterogeneous catalytic selective hydrogenation (HCSH) of VO can maximize the fraction of C18:1 in a VO bulk.^[^ ^1^ ^,^ ^2^ ^]^ HCSH can also be used to refine biodiesel.^[^ ^3^ ^]^ Selective hydrogenation offers flexibility through the use of different commodity feedstocks in existing processes in the chemical value chain, keeping competitiveness in the sustainable chemical industry.^[^ ^4^ ^]^ Moreover, in the light of Green Chemistry and Green Engineering principles, hydrogenation reactions can be advantageous: the atom economy can reach 100%; often, no solvent is required and the catalysts are recyclable or regeneratable; green hydrogen can be used for the process.^[^ ^5^ ^]^ Pt, Pd, and Ni are common metal active phases for HCSH.^[^ ^6^ ^]^

From the point of view of kinetic modeling, approaches with different complexity levels appeared in the literature for HCSH of VO.^[^ ^7^ ^–^ ^11^ ^]^ Isomerization of double bonds can be neglected by lumping C18:i according to the number of unsaturations ‘i’ (C18:3 for linolenic, C18:2 for linoleic, C18:1 for oleic, C18:0 for stearic); pseudo‐first‐order kinetic laws can be adopted for each hydrogenation step.^[^ ^9^, ^10^ ^–^ ^11^ ^]^ In addition, one can exclude ‘shunt reactions’,^[^ ^12^ ^,^ ^13^ ^]^ i.e., multiple cohydrogenations of two or more double bonds of Poly‐Unsaturated Fatty Acids (PUFA, C18:i with i ≥ 2).^[^ ^13^ ^]^

This research group previously modeled HCSH by pseudo‐first‐order kinetics,^[^ ^14^ ^]^ using data from batch HCSH tests at laboratory‐scale on canola oil and commercial Lindlar catalyst (supported‐Pd, partially poisoned):^[^ ^15^ ^]^ the experimental HCSH data could be modeled appropriately—with or without shunt reactions—only by two subsequent pseudo‐first‐order kinetic regimes, each one with its own set of kinetic constants; one was valid before maximization of C18:1, the other after this point.^[^ ^14^ ^]^ In other words, pseudo‐first‐order rate laws worked only with variable selectivity^[^ ^14^ ^]^ (defined as the ratios of pseudo‐first‐order kinetic constants^[^ ^8^ ^]^). This was attributed to different adsorptive interactions between Pd and PUFA or monounsaturated species,^[^ ^14^ ^,^ ^16^ ^]^ a situation which would require more complex kinetics interpretation.

In this work, therefore, a more in‐depth analysis of the kinetics of HCSH was carried out: the pseudo‐first‐order approach was replaced by an investigation concerning the reaction mechanisms developed according to the Hougen–Watson approach,^[^ ^17^ ^]^ extending the work of Bernas et al.^[^ ^18^ ^]^ to an actual VO mixture (i = 0, 1, 2, 3).

## 2 Materials and Methods

### 2.1 HCSH Experiments

The details of HCSH experiments and methods to identify the species in the liquid product are available elsewhere.^[^ ^15^ ^]^ For the sake of clarity, it is reminded that the HCSH tests (**Table** **1**) were performed in a 600 mL batch reactor, on commercial canola oil, at temperatures (T) of 120–180 °C, continuously insufflating H_2_ in the oil bulk at regulated pressures ($p_{H_{2}}$) of 0.4–1.2 MPa, stirring at 600 rpm (maximum allowed) to suspend the fine particles of Lindlar catalyst and disperse the H_2_ bubbles. Negligible volumes of oil samples were taken regularly until the end of each experiment ($t_{fin}$), with appropriate sampling frequencies. The relative molar fractions of lumped C18:i classes ( i = 0, 1, 2, 3) were measured off‐line for each sample by gas‐chromatographic methods detailed elsewhere.^[^ ^15^ ^]^ The Supporting Information (SI) collects the datasets of molar fractions from tests in Table 1.

**Table 1:** HCSH test conditions (adapted from[^14^, ^15^]).

	Test 3a)	Test 4a)	Test 5a)
T [°C]	120 ± 5	180 ± 5	180 ± 5
$p_{H_{2}}$ [MPa]	0.8	0.4	1.2
$t_{fin}$ [h]	6	6	5

a) Test numbering from.^[^ ^15^ ^]^

### 2.2 Modeling Hypotheses

For Hougen–Watson modeling, the following hypotheses were included in all developed mechanisms:^[^ ^18^ ^]^

1. no shunt‐reactions occur;
2. no isomerization is included, and the position of double bonds does not modify adsorption behavior; therefore, the considered species are the C18:i lumped in the four classes with i = 0, 1, 2, 3;
3. no noncatalytic hydrogenation occurs;
4. catalyst surfaces are ideal as per the Langmuir meaning;
5. surface reactive steps are irreversible; their kinetic constants ($k_{n}$, for the nth elementary step) depend on temperature (T) according to the Arrhenius equation (Equation (1), where $A_{n}$ is the pre‐exponential factor, $E_{a,n}$ is the activation energy, R is the ideal gas constant, and $T_{mean}$ is the overall average temperature of tests in Table 1);
(1) $$k_{n}=A_{n}\exp\left(-\frac{E_{a,n}}{R}\left(\frac{1}{T}-\frac{1}{T_{mean}}\right)\right)$$
6. C18:i always adsorb competitively, by unsaturations that interact with catalyst active sites (i.e., C18:0 desorption is instantaneous);
7. one active site (Z) adsorbs one unsaturated C18:i molecule (i = 1, 2, 3) or one corresponding reaction intermediate;
8. adsorption/desorption steps are at their quasiequilibria: in this work—differently from sensible simplifications of Bernas et al.^[^ ^18^ ^]^—each C18:i can have its adsorptive behavior and all adsorptions/desorptions depend on temperature; therefore, each C18:i has a peculiar adsorption/desorption equilibrium constant ($K_{m}$, for the mth elementary step), which depends on T by a van’t Hoff law (Equation (2), where $B_{m}$ is the pre‐exponential factor, $\text{Δ}H_{m}$ is the adsorption/desorption enthalpy);
(2) $$K_{m}=B_{m}\exp\left(-\frac{\text{Δ}H_{m}}{R}\left(\frac{1}{T}-\frac{1}{T_{mean}}\right)\right)$$
9. the pseudo‐steady state hypothesis was applied to the surface reaction intermediates (i.e., adsorbed species other than hydrogen or C18:i);
10. no intraparticle and interparticle (film) mass‐transfer limitations occur, i.e., observed data correspond to the intrinsic kinetically limited regime.

On the other hand, the following occurrences were varied to produce the six different mechanisms:

• H_2_ adsorption on the catalyst occurs (Hinshelwood–Langmuir) or not (Eley–Rideal);
• H_2_ adsorption is molecular or dissociative;
• H_2_ adsorption is competitive with fatty acids adsorption (on sites Z) or not‐competitive (on dedicated sites Z′);
• reaction surface‐intermediates are formed or not.

From here on, it is convenient to name C18:3 as A, C18:2 as B, C18:1 as C, and C18:0 as D.

According to hypotheses 1, 2, and 3, the overall HCSH reaction is a sequence of consecutive hydrogenations, described by Equation (3).

(3) $$A\;\overset{+H_{2}}{\to }\;B\;\overset{+H_{2}}{\to }\;C\;\overset{+H_{2}}{\to }\;D$$

Each mechanism generated its specific set of algebraic expressions for the reaction rate laws referred to A, B, C, D ($r_{i}$ with i=A,B,C,D), which contained the parameters $A_{n}$, $E_{a,n}$, $B_{m}$, and $\text{Δ}H_{m}$ to be regressed. These rate laws were introduced into the mass balances of the experimental batch reactor, constituting the system of Ordinary Differential Equation (ODE, Equation (4), with $X_{i}$ as the relative molar fractions of i=A,B,C,D, i.e., the ratio $\left(\text{moles}\;of\;i)/\sum_{i}(\text{moles}\;of\;i\right)$). The SI details the generation of reaction rate laws for each mechanism.

(4) $$\{\begin{array}{l} \frac{dX_{A}}{dt}=r_{A}\\ \frac{dX_{B}}{dt}=r_{B}\\ \frac{dX_{C}}{dt}=r_{C}\\ \frac{dX_{D}}{dt}=r_{D} \end{array}$$

### 2.3 Computational Methods

A MATLAB R2023b script was purposely developed for the nonlinear regression of the kinetic parameters $A_{n}$, $E_{a,n}$, $B_{m}$, and $\text{Δ}H_{m}$ by the least‐squares minimization method (MATLAB “lsqnonlin”), globally applied^[^ ^18^ ^]^ to the whole set of experimental data from all tests in Table 1. The objective function of this least‐squares minimization problem is the global sum of squared residuals ($SSR_{\text{global}}$), which concerned all tests, samples, and components (Equation (5)):

(5) $$SSR_{\text{global}}=\sum_{h=3,4,5}^{\text{Tests}}\sum_{j=0,\ldots ,t_{fin}}^{\text{Samples}}\sum_{i=A,B,C,D}^{\text{Components}}{\left(X_{ijh}-{\hat{X}}_{ijh}\right)}^{2}$$

where $X_{ijh}$ is the experimental value of molar fraction of the ith component, measured for jth sample of the hth test, and ${\hat{X}}_{ijh}$ is the time‐corresponding value obtained from the integration of the ODE system of Equation (4). The numerical solution of Equation (4) was evaluated by MATLAB “ode15s”.

The regression script was applied to each developed mechanism. Once the kinetic parameters were regressed, the overall explained variance (coefficient of determination, $R^{2}$) was calculated by Equation (6) (with $\bar{X}$ as the grand average of experimental $X_{ijh}$, SST as the total sum of squares). A value of $R^{2}$ close to 1 indicates a strong agreement between model predictions and experimental data.

(6) $$R^{2}=1-\frac{SSR_{\text{global}}}{SST}=1-\frac{SSR_{\text{global}}}{\sum_{h=3,4,5}^{\text{Tests}}\sum_{j=0,\ldots ,t_{fin}}^{\text{Samples}}\sum_{i=A,B,C,D}^{\text{Components}}{\left(X_{ijh}-\bar{X}\right)}^{2}}$$

The specific sum of squared residuals for each component in each test ($SSR_{i,h}$) was calculated by Equation (7), to observe the performance of globally regressed parameters for a given component i in a given experiment h.

(7) $$SSR_{i,h}=\sum_{j=0,\ldots ,t_{fin}}^{\text{Samples}}{\left(X_{ijh}-{\hat{X}}_{ijh}\right)}^{2}\;\;\;\mathrm{with}:\;i=A,B,C,D;\;h=\mathrm{Test}\;3,4,5$$

## 3 Results and Discussion

### 3.1 HCSH Mechanisms

**Scheme 1:**
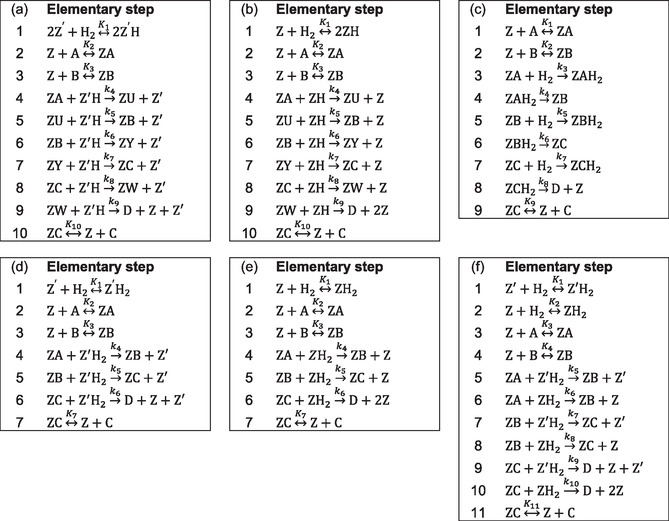
Proposed reaction mechanisms for HCSH of VO: H_2_ vs. C18:i adsorption noncompetitive,  H_2_ adsorption dissociative, with surface intermediates a); H_2_ vs. C18:i adsorption competitive H_2_ adsorption dissociative, with surface intermediates b); H_2_ not‐adsorbed (Eley–Rideal), with surface intermediates c); H_2_ vs. C18:i adsorption noncompetitive, H_2_ adsorption molecular, without surface intermediates d); H_2_ vs. C18:i adsorption competitive H_2_ adsorption molecular, without surface intermediates e); H_2_ vs. C18:i adsorption competitive and not‐competitive, H_2_ adsorption molecular, without surface intermediates f).

**Scheme** **1** summarizes the six reaction mechanisms developed under the modeling hypotheses in Section 2.2. The symbol $\overset{K_{m}}{↔}$ in Scheme 1 denotes a mth mechanism step, which is a quasi‐equilibrated adsorption/desorption with equilibrium constant $K_{m}$ Equation (2). The symbol $\overset{k_{n}}{\to }$ in Scheme 1 denotes a $n^{th}$ mechanism step, which is an irreversible surface reaction with kinetic constant $k_{n}$ Equation (1). In Scheme 1, the notation of sites Z or Z′ followed by the name of other species indicates that the species are adsorbed on Z or Z′ (e.g., ZA is A adsorbed on Z). For each mechanism in Scheme 1, the overall sum of step equations results into Equation (3).

Schemes 1a and 1b were derived by the assumption of Horiuti–Polanyi mechanism:^[^ ^18^ ^–^ ^20^ ^]^ H_2_ adsorption on Pd is dissociative and semihydrogenated surface intermediates are formed (U, Y, and W from A, B, and C, respectively); in the case of Scheme 1a, H_2_ adsorption occurs on dedicated sites Z′, thus it is not competitive with C18:i adsorption on sites Z; in the case of Scheme 1b, H_2_ and C18:i compete for adsorption on sites Z.

Scheme 1c assumes an Eley–Rideal behavior of H_2_, i.e., H_2_ directly reacts in its free molecular form with adsorbed C18:i species; adsorbed hydrogenated intermediates (e.g., ${\mathrm{AH}}_{2}$) are formed on sites Z and become adsorbed unsaturated C18:i by surface reactions.

Schemes 1d, 1e, and 1f assume molecular adsorption of H_2_ and direct hydrogenation of one unsaturation by surface reactions without intermediates; this mimics the pairwise hydrogenation of unsaturated organic molecules observed in literature.^[^ ^18^ ^,^ ^21^ ^]^ In Scheme 1d, H_2_ adsorption is not‐competitive with that of C18:i; in Scheme 1e, H_2_ adsorption is competitive with that of C18:i; in Scheme 1f, H_2_ adsorption is both competitive and not‐competitive with that of C18:i.

Equations (8), (9), (10), (11), (12), and (13) summarize the kinetic rate laws obtained for Schemes 1a, 1b, 1c, 1d, 1e, and 1f, respectively. Details of their development are summarized in the SI, Supporting Information.

(8) $$\{\begin{array}{l} r_{A}=-\frac{k_{4}K_{2}X_{A}}{DEN}\sqrt{K_{1}p_{H_{2}}}\\ r_{B}=\frac{k_{4}K_{2}X_{A}-k_{6}K_{3}X_{B}}{DEN}\sqrt{K_{1}p_{H_{2}}}\\ r_{C}=\frac{k_{6}K_{3}X_{B}-\left(\frac{k_{8}}{K_{10}}\right)X_{C}}{DEN}\sqrt{K_{1}p_{H_{2}}}\\ r_{D}=\frac{\left(\frac{k_{8}}{K_{10}}\right)X_{C}}{DEN}\sqrt{K_{1}p_{H_{2}}}\\ DEN=\left(1+K_{2}\frac{k_{5}+k_{4}}{k_{5}}X_{A}+K_{3}\frac{k_{7}+k_{6}}{k_{7}}X_{B}+\frac{1}{K_{10}}\frac{k_{9}+k_{8}}{k_{9}}X_{C}\right)\\ \left(1+\sqrt{K_{1}p_{H_{2}}}\right) \end{array}$$

(9) $$\{\begin{array}{l} r_{A}=-\frac{k_{4}K_{2}X_{A}}{DEN}\sqrt{K_{1}p_{H_{2}}}\\ r_{B}=\frac{k_{4}K_{2}X_{A}-k_{6}K_{3}X_{B}}{DEN}\sqrt{K_{1}p_{H_{2}}}\\ r_{C}=\frac{k_{6}K_{3}X_{B}-\left(\frac{k_{8}}{K_{10}}\right)X_{C}}{DEN}\sqrt{K_{1}p_{H_{2}}}\\ r_{D}=\frac{\left(\frac{k_{8}}{K_{10}}\right)X_{C}}{DEN}\sqrt{K_{1}p_{H_{2}}}\\ DEN={\left(1+K_{2}\frac{k_{5}+k_{4}}{k_{5}}X_{A}+K_{3}\frac{k_{7}+k_{6}}{k_{7}}X_{B}+\frac{1}{K_{10}}\frac{k_{9}+k_{8}}{k_{9}}X_{C}+\sqrt{K_{1}p_{H_{2}}}\right)}^{2} \end{array}$$

(10) $$\{\begin{array}{l} r_{A}=-\frac{k_{3}K_{1}X_{A}}{DEN}p_{H_{2}}\\ r_{B}=\frac{k_{3}K_{1}X_{A}-k_{5}K_{2}X_{B}}{DEN}p_{H_{2}}\\ r_{C}=\frac{k_{5}K_{2}X_{B}-\left(\frac{k_{7}}{K_{9}}\right)X_{C}}{DEN}p_{H_{2}}\\ r_{D}=\frac{\left(\frac{k_{7}}{K_{9}}\right)X_{C}}{DEN}p_{H_{2}}\\ DEN=1+K_{1}\left(\frac{k_{3}p_{H_{2}}+k_{4}}{k_{4}}\right)X_{A}+K_{2}\left(\frac{k_{5}p_{H_{2}}+k_{6}}{k_{6}}\right)X_{B}\\ +\frac{X_{C}}{K_{9}}\left(\frac{k_{7}p_{H_{2}}+k_{8}}{k_{8}}\right) \end{array}$$

(11) $$\{\begin{array}{l} r_{A}=-\frac{k_{4}K_{2}X_{A}}{DEN}K_{1}p_{H_{2}}\\ r_{B}=\frac{k_{4}K_{2}X_{A}-k_{5}K_{3}X_{B}}{DEN}K_{1}p_{H_{2}}\\ r_{C}=\frac{k_{5}K_{3}X_{B}-\left(\frac{k_{6}}{K_{7}}\right)X_{C}}{DEN}K_{1}p_{H_{2}}\\ r_{D}=\frac{\left(\frac{k_{6}}{K_{7}}\right)X_{C}}{DEN}K_{1}p_{H_{2}}\\ DEN=\left(1+K_{2}X_{A}+K_{3}X_{B}+\frac{X_{C}}{K_{7}}\right)\left(1+K_{1}p_{H_{2}}\right) \end{array}$$

(12) $$\{\begin{array}{l} r_{A}=-\frac{k_{4}K_{2}X_{A}}{DEN}K_{1}p_{H_{2}}\\ r_{B}=\frac{k_{4}K_{2}X_{A}-k_{5}K_{3}X_{B}}{DEN}K_{1}p_{H_{2}}\\ r_{C}=\frac{k_{5}K_{3}X_{B}-\left(\frac{k_{6}}{K_{7}}\right)X_{C}}{DEN}K_{1}p_{H_{2}}\\ r_{D}=\frac{\left(\frac{k_{6}}{K_{7}}\right)X_{C}}{DEN}K_{1}p_{H_{2}}\\ DEN={\left(1+K_{2}X_{A}+K_{3}X_{B}+\frac{X_{C}}{K_{7}}+K_{1}p_{H_{2}}\right)}^{2} \end{array}$$

(13) $$\{\begin{array}{l} r_{A}=-\left(\frac{k_{5}K_{3}X_{A}}{DEN1}K_{1}p_{H_{2}}+\frac{k_{6}K_{3}X_{A}}{DEN2}K_{2}p_{H_{2}}\right)\\ r_{B}=\frac{k_{5}K_{3}X_{A}-k_{7}K_{4}X_{B}}{DEN1}K_{1}p_{H_{2}}+\frac{k_{6}K_{3}X_{A}-k_{8}K_{4}X_{B}}{DEN2}K_{2}p_{H_{2}}\\ r_{C}=\frac{k_{7}K_{4}X_{B}-\left(\frac{k_{9}}{K_{11}}\right)X_{C}}{DEN1}K_{1}p_{H_{2}}+\frac{k_{8}K_{4}X_{B}-\left(\frac{k_{10}}{K_{11}}\right)X_{C}}{DEN2}K_{2}p_{H_{2}}\\ r_{D}=\frac{\left(\frac{k_{9}}{K_{11}}\right)X_{C}}{DEN1}K_{1}p_{H_{2}}+\frac{\left(\frac{k_{10}}{K_{11}}\right)X_{C}}{DEN2}K_{2}p_{H_{2}}\\ DEN1=\left(1+K_{3}X_{A}+K_{4}X_{B}+\frac{X_{C}}{K_{11}}+K_{2}p_{H_{2}}\right)\left(1+K_{1}p_{H_{2}}\right)\\ DEN2={\left(1+K_{3}X_{A}+K_{4}X_{B}+\frac{X_{C}}{K_{11}}+K_{2}p_{H_{2}}\right)}^{2} \end{array}$$

### 3.2 Nonlinear Regressions

**Figure 1:**
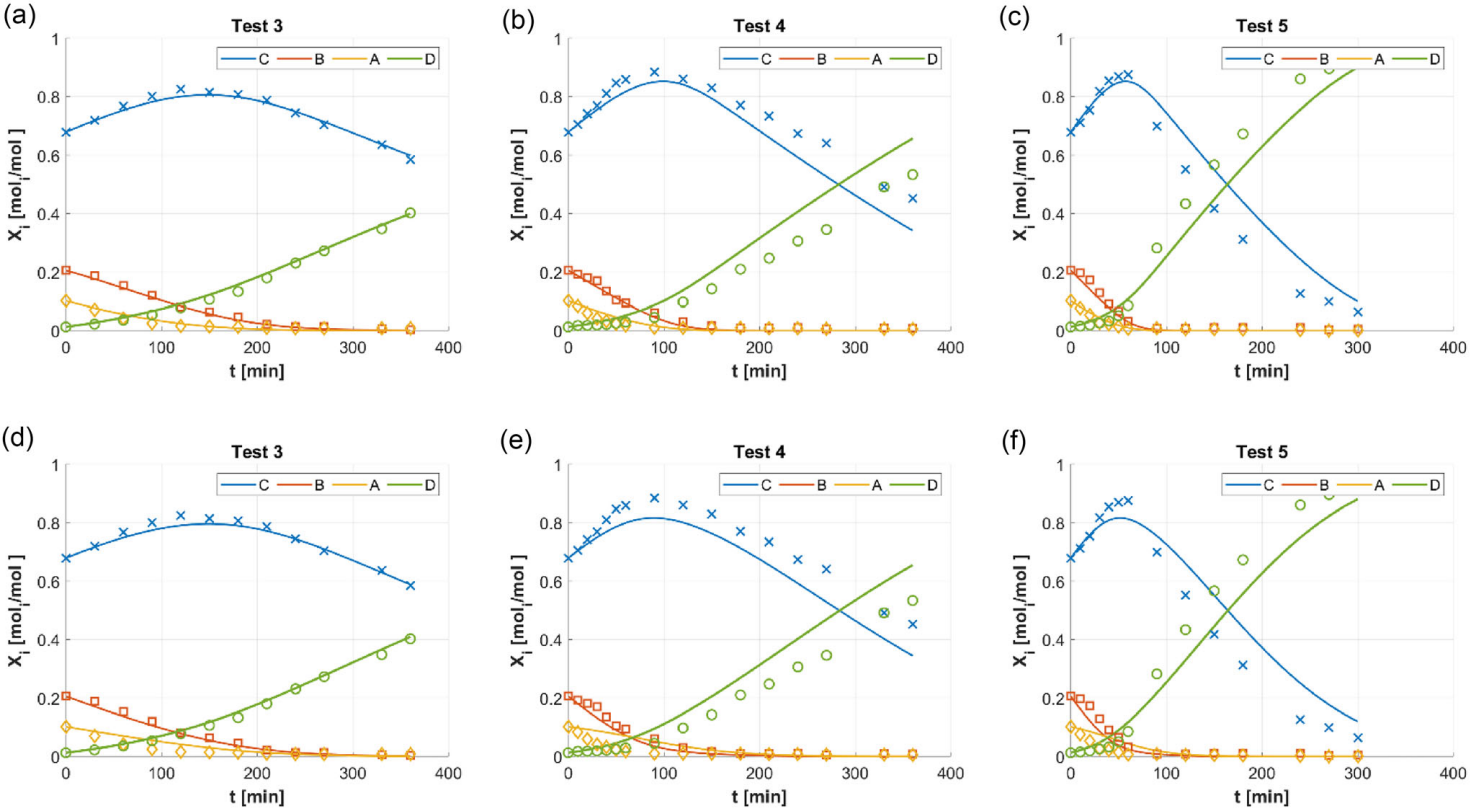
Experimental measurements of C18:i molar fractions $X_{i}$ (i=A,B,C,D) during HCSH batch tests (adapted from^[^ ^14^ ^,^ ^15^ ^]^): $X_{A}$ (C18:3, yellow ◊); $X_{B}$ (C18:2, orange □); $X_{C}$ (C18:1, blue x); $X_{D}$ (18:0, green ○). Functions ${\hat{X}}_{i}(t)$ (solid lines, same colors of experimental measurements) with parameters regressed for Scheme 1a, Equation (8): Test 3 (a), Test 4 (b), and Test 5 (c). Functions ${\hat{X}}_{i}(t)$ (solid lines, same colors of experimental measurements) with parameters regressed for Scheme 1b, Equation (9): Test 3 (d), Test 4 (e), and Test 5 (f).

**Figure 2:**
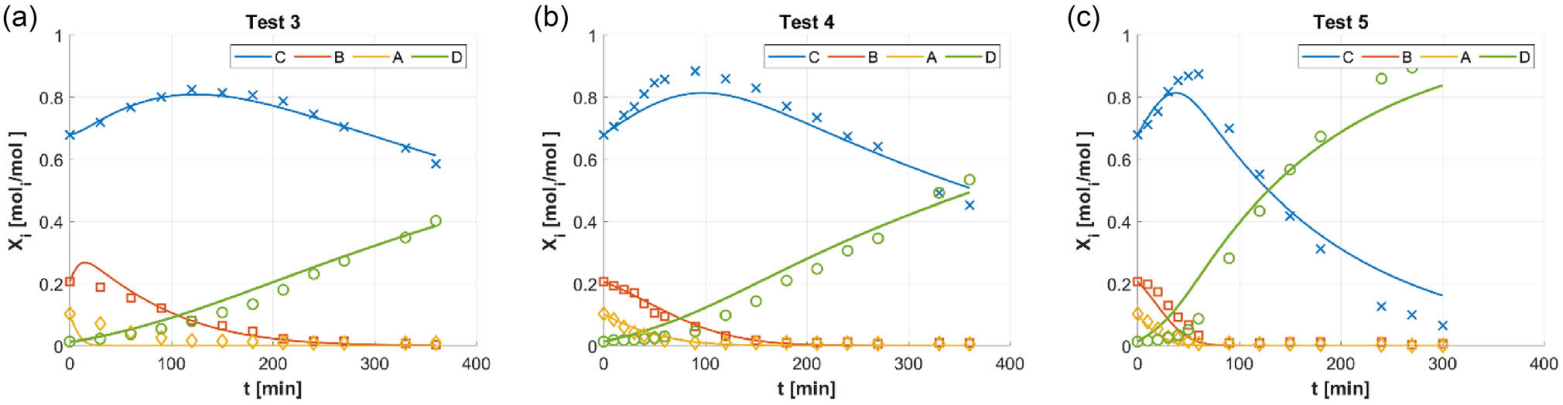
Experimental measurements of C18:i molar fractions $X_{i}$ (i=A,B,C,D) during HCSH batch tests (adapted from^[^ ^14^ ^,^ ^15^ ^]^): $X_{A}$ (C18:3, yellow ◊); $X_{B}$ (C18:2, orange □); $X_{C}$ (C18:1, blue x); $X_{D}$ (18:0, green ○;). Functions ${\hat{X}}_{i}(t)$ (solid lines, same colors of experimental measurements) with parameters regressed for Scheme 1c, Equation (10): Test 3 (a), Test 4 (b), and Test 5 (c).

**Figure 3:**
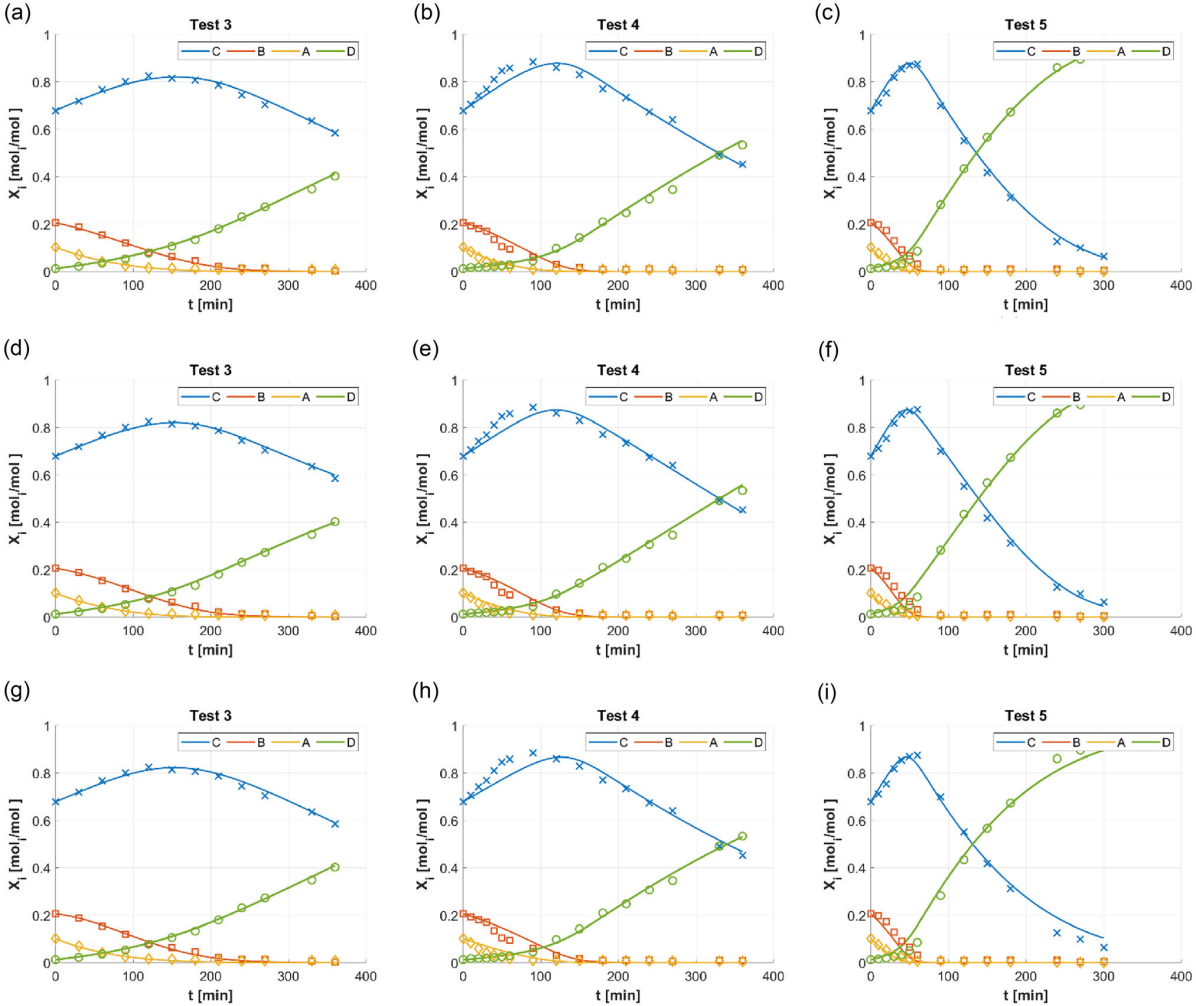
Experimental measurements of C18:i molar fractions$X_{i}$ (i=A,B,C,D) during HCSH batch tests (adapted from^[^ ^14^ ^,^ ^15^ ^]^): $X_{A}$ (C18:3, yellow ◊); $X_{B}$ (C18:2, orange □); $X_{C}$ (C18:1, blue x); $X_{D}$ (18:0, green ○;). Functions ${\hat{X}}_{i}(t)$ (solid lines, same colors of experimental measurements) with parameters regressed for Scheme 1d, Equation (11): Test 3 (a), Test 4 (b), and Test 5 (c). Functions ${\hat{X}}_{i}(t)$ (solid lines, same colors of experimental measurements) with parameters regressed for Scheme 1e, Equation (12): Test 3 (d), Test 4 (e), and Test 5 (f). Functions ${\hat{X}}_{i}(t)$ (solid lines, same colors of experimental measurements) with parameters regressed for Scheme 1f, Equation (13): Test 3 (g), Test 4 (h), and Test 5 (i).

**Figures** **1**, **2**, and **3** show the graphical results obtained by the nonlinear regressions based on different reaction mechanisms (Scheme 1) and operated on data from the tests in Table 1: the solid lines represent regressed functions of C18:i molar fractions as functions of time (${\hat{X}}_{i}(t)$), while symbols are the experimental measurements of molar fractions $X_{i}$ (i=A,B,C,D) from the samples taken during each test. **Table** **2** collects the values of the regression performance indicators $SSR_{\text{global}}$ (Equation (5)) and $R^{2}$ (Equation (6)). **Table** **3** shows the standard deviations between experimental measurements of $X_{i}$ and values of regressed curves ${\hat{X}}_{i}(t)$ at the same reaction time ($SSR_{i,h}$, Equation (7)).

**Table 2:** Regression of kinetic parameters for mechanisms in Scheme 1: rate laws, number of regressed parameters, and regression performance indicators ($SSR_{global}$, Equation (5). $R^{2}$, Equation (6).

Mechanism	Rate laws	Number of regressed parameters	$SSR_{\text{global}}$ a)	$R^{2}$
Scheme 1a	Equation (8)	20	2.77 · 10^−2^	0.942
Scheme 1b	Equation (9)	20	3.39 · 10^−1^	0.922
Scheme 1c	Equation (10)	14	2.26 · 10^−1^	0.953
Scheme 1d	Equation (11)	14	3.82 · 10^−2^	0.992
Scheme 1e	Equation (12)	18	5.04 · 10^−2^	0.989
Scheme 1f	Equation (13)	22	6.94 · 10^−2^	0.986

a) For comparison, SST in Equation (6) was 4.78·10^0^.

**Table 3:** Values of $SSR_{i,h}$ Equation (7) . For each mechanism, the first row corresponds to Test 3, the second to Test 4, and the third to Test 5.

Mechanism	$SSR_{A,h}$	$SSR_{B,h}$	$SSR_{C,h}$	$SSR_{D,h}$
Scheme 1a	0.0017a)0.0558b)0.0755c)	0.0005a)0.0018b)0.0030c)	0.0008a)0.0029b)0.0007c)	0.0018a)0.0730b)0.0596c)
Scheme 1b	0.0033a)0.0707b)0.0839c)	0.0010a)0.0047b)0.0079c)	0.0032a)0.0143b)0.0088c)	0.0013a)0.0717b)0.0679c)
Scheme 1c	0.0022a)0.0306b)0.0568c)	0.0041a)0.0013b)0.0091c)	0.0081a)0.0012b)0.0011c)	0.0081a)0.0228b)0.0809c)
Scheme 1d	0.0014a)0.0118b)0.0044c)	0.0004a)0.0037b)0.0049c)	0.0005a)0.0014b)0.0009c)	0.0007a)0.0033b)0.0049c)
Scheme 1e	0.0011a)0.0136b)0.0069c)	0.0006a)0.0037b)0.0077c)	0.0005a)0.0021b)0.0009c)	0.0008a)0.0024b)0.0103c)
Scheme 1f	0.0014a)0.0199b)0.0111c)	0.0005a)0.0051b)0.0072c)	0.0004a)0.0039b)0.0006c)	0.0005a)0.0019b)0.0169c)

a) Test 3.

b) Test 4.

c) Test 5.

Figure 1 collects the comparisons between experimental measurements and regression curves according to the mechanisms inspired by the Horiuti–Polanyi one.^[^ ^19^ ^]^ Scheme 1a was applied in Figure 1a for Test 3, Figure 1b for Test 4, and Figure 1c for Test 5; Scheme 1b was applied in Figure 1d for Test 3, Figure 1e for Test 4, and Figure 1f for Test 5. Figure 1 suggested that Schemes 1a and 1b failed to interpret the HCSH experimental phenomena; that failure became more and more evident as the severity of test conditions was increased (see Table 1). Consequently, the presence of semihydrogenated intermediates and dissociative adsorption of H_2_ did not allow the reliable representation of experimental behaviors, independently of the competition between H_2_ and C18:i fatty acids for the active sites.

Figure 2 compares experimental data and regressions related to the Eley‐Rideal hypothesis on H_2_. Scheme 1c was applied in Figure 2a for Test 3, Figure 2b for Test 4, and Figure 2c for Test 5: the regression quality worsened along with the severity of test conditions (see Table 1). This suggested that the hypothesis of Eley–Rideal for H_2_ was not suitable for the HCSH experiments considered in this work.

Figure 3 compares experimental data and regressions according to the mechanisms with molecular H_2_ adsorption. Scheme 1d was applied in Figure 3a for Test 3, Figure 3b for Test 4, and Figure 3c for Test 5; Scheme 1e was applied in Figure 3d for Test 3, Figure 3e for Test 4, and Figure 3f for Test 5; Scheme 1f was applied in Figure 3g for Test 3, Figure 3h for Test 4, and Figure 3i for Test 5. In all cases of Figure 3, the quality of regressions was better than that expressed by Figures 1 and 2, especially in the case of Test 5 (the one with more abrupt variations of fatty acid fractions). This suggested that the experiments were more adequately interpreted by the kinetic laws obtained hypothesizing molecular H_2_ adsorption and hydrogenations without reaction intermediates. Generally, the chemisorption of H_2_ on transition metals is widely recognized to be dissociative,^[^ ^22^ ^,^ ^23^ ^]^ also with solubilization in the case of Pd;^[^ ^21^ ^]^ therefore, the authors agree with Bernas et al.^[^ ^18^ ^]^ in allowing the introduction of H_2_ molecular adsorption into the mechanism only as an escamotage to simulate the pairwise addition of adsorbed ‐H to an olefinic unsaturation, a phenomenon documented for supported‐Pd by nuclear magnetic resonance spectroscopy in the literature.^[^ ^21^ ^]^

Among the generally improved performances of regressions in Figure 3, the regression performance indicators in Table 2 helped to choose the most statistically suitable mechanism for interpreting HCSH of VO. Scheme 1d was the most appropriate, thanks to its higher $R^{2}$ (Table 2); noteworthy, Scheme 1d had the lowest number of parameters (‘Occam's razor’), which made it preferable to other mechanisms with high $R^{2}$ (Table 2), such as Schemes 1e and 1f. The $SSR_{i,h}$ (Equation (7)) were collected in Table 3: the general better performance of mechanisms with molecular H_2_ adsorption (Scheme 1d–f) was confirmed, in particular for Scheme 1d.

Of all the tested mechanisms, the HCSH of VO by Lindlar catalyst was modeled with the best statistical performances by Scheme 1d. Schemes 1a and 1b gave a substantial discrepancy with experimental data (Figure 1), as also assessed by their $R^{2}$ values lower than 0.95 (Table 2). Scheme 1c deserved dedicated evaluations, as it had the lowest number of parameters, as Scheme 1d (14, Table 2): Scheme 1c had a $R^{2}$ of 0.95, slightly higher than those of Schemes 1a,b, (Table 2), but significantly lower than the $R^{2}$ of Schemes 1d–f (around 0.99, Table 2); therefore, Scheme 1c and its Eley–Rideal mechanism were not the best choice for modeling HCSH, as also confirmed by comparing Figure 2c to Figure 3c, for instance. All the models with molecular H_2_ adsorption (Schemes 1d–f) could be considered as acceptable choices, from a statistical point of view ($R^{2}$ of about 0.99, Table 2); in that case, the ‘Occam's razor’ principle became diriment, making Scheme 1d the preferred since it was the one with the lowest number of parameters; incidentally, Scheme 1d also had the highest $R^{2}$.

Noteworthy, Bernas et al.^[^ ^18^ ^]^ found that the same hypotheses of Scheme 1d gave the best rate laws to interpret their HCSH of C18:2 catalyzed by Pd/C.

### 3.3 Chemical–Physical Meaningfulness of the Statistically Best Model

Scheme 1d (Table 3) was the best mechanism to interpret HCSH of VO. It was characterized by the hypotheses that H_2_ adsorption was molecular and noncompetitive with C18:i adsorption, without formation of hydrogenation intermediates (Equation (11)). This matched well with the literature^[^ ^21^ ^]^ which reports the pairwise addition of contiguous superficial adsorbed H atoms, operated by the Pd active surface in supported catalysts. As stated in^[^ ^21^ ^]^ the dynamic isolation or localization of catalytic sites, caused by several adsorbates, can partition the metal surface into smaller regions. This could be compatible with the hypothesis of noncompetitive adsorption, with the dynamic formation of segregated active sites/ensembles of different nature.

**Table** **4** gathers the values of the parameters regressed for the kinetic laws of Scheme 1d (Equation (11)): it is worth checking their physicochemical meaningfulness. SI gathers the regressed kinetic parameters for all models, with related statistical analyses.

**Table 4:** Kinetic parameters regressed for Equation (11), i.e., rate laws of the HCSH of VO by Lindlar catalyst interpreted by the reaction mechanism in Scheme 1d.

Parameter	Units	Value	Standard deviation	[%] Standard deviation
$B_{1}$	bar_H2_^−1^	1.16 · 10^−1^	±8.66 · 10^−3^	±7.492
$\Delta H_{1}$	kJ mol^−1^	−1.01 · 10^+2^	±6.16 · 10^−3^	±0.006
$B_{2}$	mol_tot_ mol_C18:3_^−1^	1.12 · 10^+1^	±6.52 · 10^0^	±58.427
$\text{Δ}H_{2}$	kJ mol^−1^	−7.61 · 10^−1^	±4.85 · 10^−1^	±63.701
$B_{3}$	mol_tot_ mol_C18:2_^−1^	7.13 · 10^+1^	±1.77 · 10^+1^	±24.851
$\text{Δ}H_{3}$	kJ mol^−1^	−2.87 · 10^−1^	±2.61 · 10^−1^	±90.815
$B_{7}$	mol_C18:1_ mol_tot_^−1^	3.27 · 10^0^	±1.17 · 10^0^	±35.840
$\text{Δ}H_{7}$	kJ mol^−1^	+ 2.60 · 10^+1^	±1.41 · 10^0^	±5.418
$A_{4}$	mol_C18:3_ mol_tot_^−1^ min^−1^	1.22 · 10^−1^	±7.89 · 10^−2^	±64.915
$E_{a,4}$	kJ mol^−1^	5.73 · 10^+1^	±7.37 · 10^−2^	±0.129
$A_{5}$	mol_C18:2_ mol_tot_^−1^ min^−1^	1.41 · 10–02	±1.64 · 10^−3^	±11.631
$E_{a,5}$	kJ mol^−1^	5.52 · 10 + 04	±1.71 · 10^0^	±3.104
$A_{6}$	mol_C18:1_ mol_tot_^−1^ min^−1^	1.28 · 10–02	±1.63 · 10^−1^	±12.709
$E_{a,6}$	kJ mol^−1^	6.43 · 10 + 01	±1.83 · 10^0^	±2.840

The regressed adsorption enthalpy of H_2_ on the Lindlar catalyst was −101.0 kJ mol^−1^ (Table 4): this value was fully compatible with measurements on different Pd‐supported catalysts, ranging from 94 to 183 kJ mol^−1[^ ^24^ ^]^ in absolute value.

As to the adsorption of C18:i, the present study demarked different behaviors of unsaturated fatty acids when interacting with the catalyst, depending on the number of unsaturations: the PUFA (C18:2 and C18:3) had similar values of adsorption enthalpies (−0.8 and −0.3 kJ mol^−1^, respectively, Table 4), both suggesting a nearly negligible dependence on T in the investigated range; the monounsaturated C18:i had a peculiar desorption enthalpy of 26.0 kJ mol^−1^ (Table 4). By interpreting absolute values of adsorption/desorption enthalpies (i.e., heats of adsorption) as the strength of catalyst–adsorbate interaction, the modeled C18:1 had interaction energies with the solid catalyst two orders of magnitude greater than those of PUFA, which might result into an overstabilizing action within the meaning of the Sabatier principle.^[^ ^22^ ^,^ ^23^ ^]^ The values of adsorption equilibrium constants $K_{2}$, $K_{3}$, and $1/K_{7}$—calculated according to Equation (2) and Table 4—were 11.3, 71.2, and 4.8 at 120 °C, and 11.0, 70.8, and 1.7 at 180 °C; this suggested that the PUFA won the competition to accede the active sites Z at the tested conditions.

The activation energies obtained in this work (Table 4) were relatively close to those obtained by Bernas et al.^[^ ^18^ ^]^ (18.5 kJ mol^−1^ for C18:2 hydrogenation with standard error 2.3 kJ mol^−1^; 40.2 kJ mol^−1^ for C18:1 hydrogenation with standard error 3.0 kJ mol^−1^).^[^ ^18^ ^]^ The comparison between activation energies obtained in this work for the superficial hydrogenation of C18:1 (64.3 kJ mol^−1^, Table 4) and that of PUFA (57.3 and 55.4 kJ mol^−1^, Table 4) highlighted a slightly harder activation of C18:1.

All these inputs from the study of the values in Table 4 have added new details that supported what had been supposed by this research group in the previous modeling study by pseudo‐first order kinetics:^[^ ^14^ ^]^ PUFA have different affinity and adsorption strength on Pd than monounsaturated C18:1,^[^ ^16^ ^]^ so the formation of C18:1 is selectively favored in the presence of PUFA, rather than C18:1 consumption.^[^ ^16^ ^]^ In other words, the reactivity of C18:1 depends on the presence of PUFA during HCSH of VO.

## 4 Conclusion

The heterogeneous selective catalytic hydrogenation of vegetable oils was investigated kinetically by developing tailored mechanisms and related rate laws, for the case of rapeseed oil treated with a commercial Lindlar catalyst (supported‐Pd, partially poisoned), in the ranges of 120–180 °C and 0.4–1.2 MPa of H_2_. Rate laws were developed for six different reaction mechanisms and nonlinear regressions of their kinetic parameters were performed: the mechanism which simulated molecular and noncompetitive adsorption of H_2_, without reaction surface intermediates, was evaluated as the best for interpreting the experimental data. This mechanism may correspond to the pairwise hydrogenation of unsaturated organic molecules operated by Pd‐supported catalysts, observed in the literature. The values of obtained kinetic parameters are physically–chemically meaningful. This discourages the application of the more traditional Horiuti–Polanyi mechanism to the tested situation. Overall, the usage of the Hougen–Watson kinetic modeling approach was worthy to be applied to the case study, as it allowed for more precise interpretations of selectivity during HCSHs of a real vegetable oil, going beyond the inferences allowed by pseudo‐first‐order kinetics. This work provided a fully predictive modeling approach and tool, to frame the maximization of monounsaturated fatty acid fraction, valuable for industrial scale‐up studies.

## Conflict of Interest

The authors declare no conflict of interest.

## Supporting information

Supplementary Material

## Data Availability

The data that support the findings of this study are available in the supplementary material of this article.
