# Supplementary material for: Batch Heterogeneous Catalytic Selective Hydrogenation of Vegetable Oils Over Lindlar Catalyst: Kinetic Modeling Supported by Reaction Mechanisms
Source: ChemistryOpen. 2025 Sep 24;14(12):e202500369. doi: 10.1002/open.202500369 (PMC12680578; doi:10.1002/open.202500369)
Supplement: Supplementary file 1 — Supplementary Material [file OPEN-14-e202500369-s001.pdf]

SUPPORTING INFORMATION

Batch Heterogeneous Catalytic Selective Hydrogenation of Vegetable Oils Over Lindlar Catalyst: Kinetic Modelling Supported by Reaction Mechanisms.

Enza Pellegrino [a], Katia Gallucci [a], Nicoletta Cancrini [a] and Andrea Di Giuliano*[a]

“Data! data! data!…I can't make bricks without clay”
(The Adventures of Sherlock Holmes (1892) ‘The Adventure of the Copper Beeches’)
Arthur Conan Doyle 1859–1930
Scottish-born writer of detective fiction

[a] E. Pellegrino, K. Gallucci, N. Cancrini, A. Di Giuliano
Department of Industrial and Information Engineering and Economics
Università degli Studi dell’Aquila
Piazzale E. Pontieri 1 loc. Monteluco di Roio, 67100 L’Aquila (AQ), Italy
E-mail: andrea.digiuliano@univaq.it

WILEY-VCH

Experimental datasets of relative molar fractions

Table S.1. Experimental results of Test 3 in terms of relative fractions of the different fatty acids of interest: Lindlar catalyst; canola oil; 120 °C; 0.8 MPa; 4 $mg_{catalyst}/mL_{oil}$ (adapted from [10,11])

t (time) [min]	X_D (C18:0) [mol_D/mol]	X_C (C18:1) [mol_C/mol]	X_B (C18:2) [mol_B/mol]	X_A (C18:3) [mol_A/mol]
0	0.0129	0.6794	0.2056	0.1020
30	0.0223	0.7198	0.1883	0.0696
60	0.0363	0.7682	0.1538	0.0417
90	0.0538	0.8019	0.1197	0.0246
120	0.0781	0.8258	0.0804	0.0157
150	0.1064	0.8148	0.0644	0.0143
180	0.1330	0.8079	0.0461	0.0130
210	0.1797	0.7874	0.0226	0.0102
240	0.2307	0.7461	0.0145	0.0088
270	0.2728	0.7052	0.0137	0.0084
330	0.3483	0.6373	0.0062	0.0082
360	0.4026	0.5861	0.0035	0.0079

WILEY-VCH

Table S.2. Experimental results of Test 4 in terms of relative fractions of the different fatty acids of interest: Lindlar catalyst; canola oil; 180 °C; 0.4 MPa; 4 $mg_{catalyst}/mL_{oil}$ (adapted from [10,11])

t **(time) [min]**	X_D **(C18:0) [mol$_D$/mol]**	X_C **(C18:1) [mol$_C$/mol]**	X_B **(C18:2) [mol$_B$/mol]**	X_A **(C18:3) [mol$_A$/mol]**
0	0.0129	0.6794	0.2056	0.1020
10	0.0178	0.7065	0.1924	0.0833
20	0.0181	0.7423	0.1798	0.0599
30	0.0191	0.7693	0.1695	0.0422
40	0.0213	0.8106	0.1350	0.0332
50	0.0252	0.8465	0.1049	0.0234
60	0.0296	0.8589	0.0947	0.0168
90	0.0452	0.8838	0.0609	0.0101
120	0.0977	0.8608	0.0316	0.0100
150	0.1427	0.8300	0.0175	0.0098
180	0.2097	0.7709	0.0103	0.0091
210	0.2474	0.7347	0.0094	0.0085
240	0.3061	0.6748	0.0113	0.0079
270	0.3458	0.6417	0.0053	0.0072
330	0.4917	0.4923	0.0098	0.0063
360	0.5339	0.4527	0.0080	0.0053

WILEY-VCH

Table S.3. Experimental results of Test 5 in terms of relative fractions of the different fatty acids of interest: Lindlar catalyst; canola oil; 180 °C; 1.2 MPa; 4 $mg_{catalyst}/mL_{oil}$ (adapted from [10,11])

t **(time)** **[min]**	X_D **(C18:0)** **[mol_D/mol]**	X_C **(C18:1)** **[mol_C/mol]**	X_B **(C18:2)** **[mol_B/mol]**	X_A **(C18:3)** **[mol_A/mol]**
0	0.0129	0.6794	0.2056	0.1020
10	0.0155	0.7118	0.1965	0.0762
20	0.0191	0.7542	0.1733	0.0534
30	0.0260	0.8179	0.1298	0.0262
40	0.0332	0.8536	0.0910	0.0222
50	0.0510	0.8688	0.0657	0.0145
60	0.0857	0.8748	0.0322	0.0074
90	0.2820	0.7009	0.0084	0.0087
120	0.4340	0.5518	0.0082	0.0060
150	0.5670	0.4171	0.0109	0.0050
180	0.6737	0.3113	0.0109	0.0041
240	0.8605	0.1263	0.0115	0.0016
270	0.8961	0.0982	0.0042	0.0015
300	0.9283	0.0649	0.0057	0.0011

WILEY-VCH

Development of rate equations

Symbols in reaction mechanisms

Z	active site for C18:i adsorption with i = 1, 2, 3 (0 does not adsorb) and H_2 adsorption in competitive cases
Z'	active site for H_2 adsorption in non-competitive cases
	H_2 dissolved hydrogen
A	C18:3
B	C18:2
C	C18:1
D	C18:0
U	semi-hydrogenated A, surface reaction intermediate
Y	semi-hydrogenated B, surface reaction intermediate
W	semi-hydrogenated C, surface reaction intermediate
AH_2	hydrogenated A, surface reaction intermediate
BH_2	hydrogenated B, surface reaction intermediate
CH_2	hydrogenated C, surface reaction intermediate
Zq	species q adsorbed on Z
$Z'q$	species q adsorbed on Z'

Symbols in equations

X_i	relative molar fraction of C18:i
t	reaction time
t_{fin}	reaction final time
T	absolute temperature [K]
T_{mean}	average temperature of Tests 3, 4 ,5 [K]
r_i	reaction rate referred to $i = \mathrm{A,B,C,D}$
R	ideal gas constant, 8.314(462618) J mol^{-1} K^{-1}
p_{H_2}	regulated partial pressure of hydrogen
θ_q	fraction of active sites Z occupied by q ; fraction of free active sites Z if $q = 0$
θ'_q	fraction of active sites Z′ occupied by q ; fraction of free active sites Z′ if $q = 0$
k_n	kinetic constant of the nth elementary step (irreversible surface reaction)
A_n	pre-exponential factor of Arrhenius expression for k_n (Equation 1)
$E_{a,n}$	activation energy of Arrhenius expression for k_n (Equation 1)
K_m	equilibrium constant of the mth elementary step (quasi-equilibrium adsorption/desorption)
B_m	pre-exponential factor of van't Hoff expression for K_m (Equation 2)
ΔH_m	variation of adsorption/desorption enthalpy in van't Hoff expression for K_m (Equation 2)

$$k_n = A_n \exp\left(-\frac{E_{a,n}}{R}\left(\frac{1}{T} - \frac{1}{T_{mean}}\right)\right) \qquad \textit{Equation 1}$$

$$K_m = B_m \exp\left(-\frac{\Delta H_n}{R}\left(\frac{1}{T} - \frac{1}{T_{mean}}\right)\right) \qquad \textit{Equation 2}$$

WILEY-VCH

Scheme 1(a)

Elementary steps	Basic routes N(1)	N(2)	N(3)
1) $2Z' + H_2 \overset{K_1}{\leftrightarrow} 2Z'H$	1	1	1
2) $Z + A \overset{K_2}{\leftrightarrow} ZA$	1	0	0
3) $Z + B \overset{K_3}{\leftrightarrow} ZB$	-1	1	0
4) $ZA + Z'H \overset{k_4}{\rightarrow} ZU + Z'$	1	0	0
5) $ZU + Z'H \overset{k_5}{\rightarrow} ZB + Z'$	1	0	0
6) $ZB + Z'H \overset{k_6}{\rightarrow} ZY + Z'$	0	1	0
7) $ZY + Z'H \overset{k_7}{\rightarrow} ZC + Z'$	0	1	0
8) $ZC + Z'H \overset{k_8}{\rightarrow} ZW + Z'$	0	0	1
9) $ZW + Z'H \overset{k_9}{\rightarrow} D + Z + Z'$	0	0	1
10) $ZC \overset{K_{10}}{\leftrightarrow} Z + C$	0	1	-1
	$A \xrightarrow{+H_2} B$	$B \xrightarrow{+H_2} C$	$C \xrightarrow{+H_2} D$

Rates of reactions $r_A,\ r_B,\ r_C,\ r_D$ are related to rates of irreversible surface irreversible reactions (elementary steps) as detailed in Equation S. 1.

$$\begin{cases} r_{\mathrm{A}} = -r_4 \\ r_B = r_5 - r_6 \\ r_C = r_7 - r_8 \\ r_D = r_9 \end{cases}$$ *Equation S. 1*

Rates of irreversible surface irreversible reactions (elementary steps) are in Equation S. 2.

$$\begin{cases} r_4 = k_4\theta_{\mathrm{A}}\theta'_{\mathrm{H}} \\ r_5 = k_5\theta_{\mathrm{U}}\theta'_{\mathrm{H}} \\ r_6 = k_6\theta_{\mathrm{B}}\theta'_{\mathrm{H}} \\ r_7 = k_7\theta_{\mathrm{Y}}\theta'_{\mathrm{H}} \\ r_8 = k_8\theta_C\theta'_{\mathrm{H}} \\ r_9 = k_9\theta_W\theta'_{\mathrm{H}} \end{cases}$$ *Equation S. 2*

Equilibrium constants of adsorption/desorption steps are in Equation S. 3, from which expressions in Equation S. 4 can be obtained.

$$\begin{cases} K_1 = \dfrac{(\theta'_{\mathrm{H}})^2}{p_{H_2}(1-\theta'_{\mathrm{H}})^2} \\ K_2 = \dfrac{\theta_{\mathrm{A}}}{X_{\mathrm{A}}\ \theta_0} \\ K_3 = \dfrac{\theta_{\mathrm{B}}}{X_{\mathrm{B}}\ \theta_0} \\ K_{10} = \dfrac{X_{\mathrm{C}}\ \theta_0}{\theta_{\mathrm{C}}} \end{cases}$$ *Equation S. 3*

$$\begin{cases} \theta'_{\mathrm{H}} = \dfrac{\sqrt{K_1 p_{H_2}}}{1+\sqrt{K_1 p_{H_2}}} \\ \theta_{\mathrm{A}} = K_2\theta_0 X_{\mathrm{A}} \\ \theta_{\mathrm{B}} = K_3\theta_0 X_{\mathrm{B}} \\ \theta_{\mathrm{C}} = \dfrac{X_{\mathrm{C}}\ \theta_0}{K_{10}} \end{cases}$$ *Equation S. 4*

WILEY-VCH

The Pseudo-Steady-State-Hypothesis (PSSH) is applied to reaction intermediates $\mathrm{U}, \mathrm{Y}, \mathrm{W}$, imposing that $r_4 = r_5$, $r_6 = r_7$, $r_8 = r_9$, respectively; therefore, according to Equation S. 2, one obtains the Equation S. 5:

$$\begin{cases} \theta_{\mathrm{U}} = \dfrac{k_4}{k_5}\theta_{\mathrm{A}} \\ \theta_{\mathrm{Y}} = \dfrac{k_6}{k_7}\theta_{\mathrm{B}} \\ \theta_{W} = \dfrac{k_8}{k_9}\theta_{C} \end{cases}$$ *Equation S. 5*

The balance on sites Z is in Equation S. 6. The balance on sites Z' is already implied in Equation S. 3 ($1 = \theta_0' + \theta_{\mathrm{H}}'$).

$$1 = \theta_0 + \theta_{\mathrm{A}} + \theta_{\mathrm{U}} + \theta_{\mathrm{B}} + \theta_{\mathrm{Y}} + \theta_{\mathrm{C}} + \theta_{\mathrm{W}}$$ *Equation S. 6*

By substituting Equation S. 4 and Equation S. 5 in Equation S. 6, one obtains the expression of θ_0 in Equation S. 7.

$$\theta_0 = \frac{1}{1 + K_2\dfrac{k_5 + k_4}{k_5}X_{\mathrm{A}} + K_3\dfrac{k_7 + k_6}{k_7}X_{\mathrm{B}} + \dfrac{1}{K_{10}}\dfrac{k_9 + k_8}{k_9}X_{\mathrm{C}}}$$ *Equation S. 7*

By substituting Equation S. 2, Equation S. 4, Equation S. 5, and Equation S. 7 in Equation S. 1, the expressions of reaction rates are obtained as functions of observable X_i for Scheme 1(a) (Equation 8).

$$\begin{cases} r_{\mathrm{A}} = -\dfrac{k_4\,K_2\,X_{\mathrm{A}}}{DEN}\sqrt{K_1 p_{H_2}} \\ r_{\mathrm{B}} = \dfrac{k_4\,K_2\,X_{\mathrm{A}} - k_6\,K_3\,X_{\mathrm{B}}}{DEN}\sqrt{K_1 p_{H_2}} \\ r_{\mathrm{C}} = \dfrac{k_6\,K_3\,X_{\mathrm{B}} - \left(\dfrac{k_8}{K_{10}}\right)X_{\mathrm{C}}}{DEN}\sqrt{K_1 p_{H_2}} \\ r_{\mathrm{D}} = \dfrac{\left(\dfrac{k_8}{K_{10}}\right)X_{\mathrm{C}}}{DEN}\sqrt{K_1 p_{H_2}} \\ DEN = \left(1 + K_2\dfrac{k_5 + k_4}{k_5}X_{\mathrm{A}} + K_3\dfrac{k_7 + k_6}{k_7}X_{\mathrm{B}} + \dfrac{1}{K_{10}}\dfrac{k_9 + k_8}{k_9}X_{\mathrm{C}}\right)\left(1 + \sqrt{K_1 p_{H_2}}\right) \end{cases}$$ *Equation 8*

20 parameters must be regressed for Scheme 1(a):

- A_n, $E_{a,n}$ with $n = 4, 5, 6, 7, 8, 9$
- B_m, ΔH_m with $n = 1, 2, 3, 10$

Scheme 1(b)

Elementary steps	Basic routes N(1)	N(2)	N(3)
1) $Z + H_2 \overset{K_1}{\leftrightarrow} 2ZH$	1	1	1
2) $Z + A \overset{K_2}{\leftrightarrow} ZA$	1	0	0
3) $Z + B \overset{K_3}{\leftrightarrow} ZB$	-1	1	0
4) $ZA + ZH \overset{k_4}{\rightarrow} ZU + Z$	1	0	0
5) $ZU + ZH \overset{k_5}{\rightarrow} ZB + Z$	1	0	0
6) $ZB + ZH \overset{k_6}{\rightarrow} ZY + Z$	0	1	0
7) $ZY + ZH \overset{k_7}{\rightarrow} ZC + Z$	0	1	0
8) $ZC + ZH \overset{k_8}{\rightarrow} ZW + Z$	0	0	1
9) $ZW + ZH \overset{k_9}{\rightarrow} D + 2Z$	0	0	1
10) $ZC \overset{K_{10}}{\leftrightarrow} Z + C$	0	1	-1
	$A \xrightarrow{+H_2} B$	$B \xrightarrow{+H_2} C$	$C \xrightarrow{+H_2} D$

Rates of reactions $r_A,\ r_B,\ r_C,\ r_D$ are related to rates of irreversible surface irreversible reactions (elementary steps) as detailed in Equation S. 8.

$$\begin{cases} r_A = -r_4 \\ r_B = r_5 - r_6 \\ r_C = r_7 - r_8 \\ r_D = r_9 \end{cases}$$ *Equation S. 8*

Rates of irreversible surface irreversible reactions (elementary steps) are in Equation S. 9.

$$\begin{cases} r_4 = k_4\theta_A\theta_H \\ r_5 = k_5\theta_U\theta_H \\ r_6 = k_6\theta_B\theta_H \\ r_7 = k_7\theta_Y\theta_H \\ r_8 = k_8\theta_C\theta_H \\ r_9 = k_9\theta_W\theta_H \end{cases}$$ *Equation S. 9*

Equilibrium constants of adsorption/desorption steps are in Equation S. 10, from which expressions in Equation S. 11 can be obtained.

$$\begin{cases} K_1 = \dfrac{\theta_H^2}{p_{H_2}\ \theta_0^2} \\ K_2 = \dfrac{\theta_A}{X_A\ \theta_0} \\ K_3 = \dfrac{\theta_B}{X_B\ \theta_0} \\ K_{10} = \dfrac{X_C\ \theta_0}{\theta_C} \end{cases}$$ *Equation S. 10*

$$\begin{cases} \theta_H = \theta_0\sqrt{K_1 p_{H_2}} \\ \theta_A = K_2\theta_0 X_A \\ \theta_B = K_3\theta_0 X_B \\ \theta_C = \dfrac{X_C\ \theta_0}{K_{10}} \end{cases}$$ *Equation S. 11*

The Pseudo-Steady-State-Hypothesis (PSSH) is applied to reaction intermediates $\mathrm{U}, \mathrm{Y}, \mathrm{W}$, imposing that $r_4 = r_5$, $r_6 = r_7$, $r_8 = r_9$, respectively; therefore, according to Equation S. 9, one obtains the Equation S. 12:

$$\begin{cases} \theta_{\mathrm{U}} = \dfrac{k_4}{k_5}\theta_{\mathrm{A}} \\ \theta_{\mathrm{Y}} = \dfrac{k_6}{k_7}\theta_{\mathrm{B}} \\ \theta_W = \dfrac{k_8}{k_9}\theta_C \end{cases}$$

Equation S. 12

The balance on sites Z is in *Equation S. 13*.

$$1 = \theta_0 + \theta_{\mathrm{H}} + \theta_{\mathrm{A}} + \theta_{\mathrm{U}} + \theta_{\mathrm{B}} + \theta_{\mathrm{Y}} + \theta_{\mathrm{C}} + \theta_{\mathrm{W}}$$

Equation S. 13

By substituting Equation S. 11 and Equation S. 12 in Equation S. 13, one obtains the expression of θ_0 in Equation S. 14.

$$\theta_0 = \frac{1}{1 + K_2\dfrac{k_5 + k_4}{k_5}X_{\mathrm{A}} + K_3\dfrac{k_7 + k_6}{k_7}X_{\mathrm{B}} + \dfrac{1}{K_{10}}\dfrac{k_9 + k_8}{k_9}X_{\mathrm{C}} + \sqrt{K_1 p_{H_2}}}$$

Equation S. 14

By substituting Equation S. 11, Equation S. 12, Equation S. 14, and Equation S. 9 in Equation S. 8, the expressions of reaction rates are obtained as functions of observable X_i for Scheme 1(b) (Equation 9).

$$\begin{cases} r_{\mathrm{A}} = -\dfrac{k_4\, K_2\, X_{\mathrm{A}}}{DEN}\sqrt{K_1 p_{H_2}} \\ r_{\mathrm{B}} = \dfrac{k_4\, K_2\, X_{\mathrm{A}} - k_6\, K_3\, X_{\mathrm{B}}}{DEN}\sqrt{K_1 p_{H_2}} \\ r_{\mathrm{C}} = \dfrac{k_6\, K_3\, X_{\mathrm{B}} - \left(\dfrac{k_8}{K_{10}}\right)X_{\mathrm{C}}}{DEN}\sqrt{K_1 p_{H_2}} \\ r_{\mathrm{D}} = \dfrac{\left(\dfrac{k_8}{K_{10}}\right)X_{\mathrm{C}}}{DEN}\sqrt{K_1 p_{H_2}} \\ DEN = \left(1 + K_2\dfrac{k_5 + k_4}{k_5}X_{\mathrm{A}} + K_3\dfrac{k_7 + k_6}{k_7}X_{\mathrm{B}} + \dfrac{1}{K_{10}}\dfrac{k_9 + k_8}{k_9}X_{\mathrm{C}} + \sqrt{K_1 p_{H_2}}\right)^2 \end{cases}$$

Equation 9

20 parameters must be regressed for Scheme 1(b):

- A_n, $E_{a,n}$ with $n = 4, 5, 6, 7, 8, 9$
- B_m, ΔH_m with $n = 1, 2, 3, 10$

WILEY-VCH

Scheme 1(c)

Elementary steps	N(1)	N(2)	N(3)
	**Basic routes**		
1) $Z + A \overset{K_1}{\leftrightarrow} ZA$	1	0	0
2) $Z + B \overset{K_2}{\leftrightarrow} ZB$	-1	1	0
3) $ZA + H_2 \overset{k_3}{\rightarrow} ZAH_2$	1	0	0
4) $ZAH_2 \overset{k_4}{\rightarrow} ZB$	1	0	0
5) $ZB + H_2 \overset{k_5}{\rightarrow} ZBH_2$	0	1	0
6) $ZBH_2 \overset{k_6}{\rightarrow} ZC$	0	1	0
7) $ZC + H_2 \overset{k_7}{\rightarrow} ZCH_2$	0	0	1
8) $ZCH_2 \overset{k_8}{\rightarrow} D + Z$	0	0	1
9) $ZC \overset{K_9}{\leftrightarrow} Z + C$	0	1	-1
	$A \xrightarrow{+H_2} B$	$B \xrightarrow{+H_2} C$	$C \xrightarrow{+H_2} D$

Rates of reactions $r_A,\ r_B,\ r_C,\ r_D$ are related to rates of irreversible surface irreversible reactions (elementary steps) as detailed in Equation S. 15.

$$\begin{cases} r_A = -r_3 \\ r_B = r_4 - r_5 \\ r_C = r_6 - r_7 \\ r_D = r_8 \end{cases}$$ *Equation S. 15*

Rates of irreversible surface irreversible reactions (elementary steps) are in Equation S. 16.

$$\begin{cases} r_3 = k_3\theta_A p_{H_2} \\ r_4 = k_4\theta_{AH_2} \\ r_5 = k_5\theta_B p_{H_2} \\ r_6 = k_6\theta_{BH_2} \\ r_7 = k_7\theta_C p_{H_2} \\ r_8 = k_8\theta_{CH_2} \end{cases}$$ *Equation S. 16*

Equilibrium constants of adsorption/desorption steps are in Equation S. 17, from which expressions in Equation S. 18 can be obtained.

$$\begin{cases} K_1 = \dfrac{\theta_A}{X_A\,\theta_0} \\ K_2 = \dfrac{\theta_B}{X_B\,\theta_0} \\ K_9 = \dfrac{X_C\,\theta_0}{\theta_C} \end{cases}$$ *Equation S. 17*

$$\begin{cases} \theta_A = K_1\theta_0 X_A \\ \theta_B = K_2\theta_0 X_B \\ \theta_C = \dfrac{X_C\,\theta_0}{K_9} \end{cases}$$ *Equation S. 18*

The Pseudo-Steady-State-Hypothesis (PSSH) is applied to reaction intermediates U, Y, W, imposing that $r_3 = r_4$, $r_5 = r_6,\ r_7 = r_8$, respectively; therefore, according to Equation S. 16, one obtains the Equation S. 19:

$$\begin{cases} \theta_{\mathrm{AH_2}} = \dfrac{k_3}{k_4}\theta_{\mathrm{A}} p_{H_2} \\ \theta_{\mathrm{BH_2}} = \dfrac{k_5}{k_6}\theta_{\mathrm{B}} p_{H_2} \\ \theta_{\mathrm{CH_2}} = \dfrac{k_7}{k_8}\theta_{\mathrm{C}} p_{H_2} \end{cases}$$ *Equation S. 19*

The balance on sites Z is in Equation S. 20.

$$1 = \theta_0 + \theta_{\mathrm{A}} + \theta_{\mathrm{AH_2}} + \theta_{\mathrm{B}} + \theta_{\mathrm{BH_2}} + \theta_{\mathrm{C}} + \theta_{\mathrm{CH_2}}$$ *Equation S. 20*

By substituting Equation S. 18 and Equation S. 19 in Equation S. 20, one obtains the expression of θ_0 in Equation S. 21.

$$\theta_0 = \frac{1}{1 + K_1\left(\dfrac{k_3 p_{H_2} + k_4}{k_4}\right)X_{\mathrm{A}} + K_2\left(\dfrac{k_5 p_{H_2} + k_6}{k_6}\right)X_{\mathrm{B}} + \dfrac{X_{\mathrm{C}}}{K_9}\left(\dfrac{k_7 p_{H_2} + k_8}{k_8}\right)}$$ *Equation S. 21*

By substituting Equation S. 18, Equation S. 19, Equation S. 21, and Equation S. 16 in Equation S. 15, the expressions of reaction rates are obtained as functions of observable X_i for Scheme 1(c) (Equation 10).

$$\begin{cases} r_{\mathrm{A}} = -\dfrac{k_3\, K_1\, X_{\mathrm{A}}}{DEN} p_{H_2} \\ r_{\mathrm{B}} = \dfrac{k_3\, K_1\, X_{\mathrm{A}} - k_5\, K_2\, X_{\mathrm{B}}}{DEN} p_{H_2} \\ r_{\mathrm{C}} = \dfrac{k_5\, K_2\, X_{\mathrm{B}} - \left(\dfrac{k_7}{K_9}\right) X_{\mathrm{C}}}{DEN} p_{H_2} \\ r_{\mathrm{D}} = \dfrac{\left(\dfrac{k_7}{K_9}\right) X_{\mathrm{C}}}{DEN} p_{H_2} \\ DEN = 1 + K_1\left(\dfrac{k_3 p_{H_2} + k_4}{k_4}\right)X_{\mathrm{A}} + K_2\left(\dfrac{k_5 p_{H_2} + k_6}{k_6}\right)X_{\mathrm{B}} + \dfrac{X_{\mathrm{C}}}{K_9}\left(\dfrac{k_7 p_{H_2} + k_8}{k_8}\right) \end{cases}$$ *Equation 10*

18 parameters must be regressed for Scheme 1(c):

- A_n, $E_{a,n}$ with $n = 3, 4, 5, 6, 7, 8$
- B_m, ΔH_m with $n = 1, 2, 9$

WILEY-VCH

Scheme 1(d)

Elementary steps	Basic routes N(1)	N(2)	N(3)
1) $Z' + H_2 \overset{K_1}{\leftrightarrow} Z'H_2$	1	1	1
2) $Z + A \overset{K_2}{\leftrightarrow} ZA$	1	0	0
3) $Z + B \overset{K_3}{\leftrightarrow} ZB$	-1	1	0
4) $ZA + Z'H_2 \overset{k_4}{\rightarrow} ZB + Z'$	1	0	0
5) $ZB + Z'H_2 \overset{k_5}{\rightarrow} ZC + Z'$	0	1	0
6) $ZC + Z'H_2 \overset{k_6}{\rightarrow} D + Z + Z'$	0	0	1
7) $ZC \overset{K_7}{\leftrightarrow} Z + C$	0	1	-1
	$A \xrightarrow{+H_2} B$	$B \xrightarrow{+H_2} C$	$C \xrightarrow{+H_2} D$

Rates of reactions $r_A,\ r_B,\ r_C,\ r_D$ are related to rates of irreversible surface irreversible reactions (elementary steps) as detailed in Equation S. 22.

$$\begin{cases} r_A = -r_4 \\ r_B = r_4 - r_5 \\ r_C = r_5 - r_6 \\ r_D = r_6 \end{cases}$$ *Equation S. 22*

Rates of irreversible surface irreversible reactions (elementary steps) are in Equation S. 23.

$$\begin{cases} r_4 = k_4\theta_A\theta'_{H_2} \\ r_5 = k_5\theta_B\theta'_{H_2} \\ r_6 = k_6\theta_C\theta'_{H_2} \end{cases}$$ *Equation S. 23*

Equilibrium constants of adsorption/desorption steps are in Equation S. 24, from which expressions in Equation S. 25 can be obtained.

$$\begin{cases} K_1 = \dfrac{\theta'_{H_2}}{p_{H_2}\left(1-\theta'_{H_2}\right)} \\ K_2 = \dfrac{\theta_A}{X_A\,\theta_0} \\ K_3 = \dfrac{\theta_B}{X_B\,\theta_0} \\ K_7 = \dfrac{X_C\,\theta_0}{\theta_C} \end{cases}$$ *Equation S. 24*

$$\begin{cases} \theta'_{H_2} = \dfrac{K_1 p_{H_2}}{1 + K_1 p_{H_2}} \\ \theta_A = K_2\theta_0 X_A \\ \theta_B = K_3\theta_0 X_B \\ \theta_C = \dfrac{X_C\,\theta_0}{K_7} \end{cases}$$ *Equation S. 25*

The balance on sites Z is in Equation S. 26. The balance on sites Z′ is already implied in Equation S. 24 ($1 = \theta'_0 + \theta'_{H_2}$).

WILEY-VCH

$$1 = \theta_0 + \theta_\mathrm{A} + \theta_\mathrm{B} + \theta_\mathrm{C}$$ *Equation S. 26*

By substituting Equation S. 25 in Equation S. 26, one obtains the expression of θ_0 in Equation S. 27.

$$\theta_0 = \frac{1}{1 + K_2 X_\mathrm{A} + K_3 X_\mathrm{B} + \frac{X_\mathrm{C}}{K_7}}$$ *Equation S. 27*

By substituting Equation S. 25, Equation S. 27, and Equation S. 23 in Equation S. 22, the expressions of reaction rates are obtained as functions of observable X_i for Scheme 1(d) (Equation 11).

$$\begin{cases} r_\mathrm{A} = -\frac{k_4\ K_2\ X_\mathrm{A}}{DEN}\ K_1 p_{H_2} \\ r_\mathrm{B} = \frac{k_4\ K_2\ X_\mathrm{A}\ - k_5\ K_3\ X_\mathrm{B}}{DEN}\ K_1 p_{H_2} \\ r_\mathrm{C} = \frac{k_5\ K_3\ X_\mathrm{B}\ - \left(\frac{k_6}{K_7}\right) X_\mathrm{C}}{DEN}\ K_1 p_{H_2} \\ r_\mathrm{D} = \frac{\left(\frac{k_6}{K_7}\right) X_\mathrm{C}}{DEN}\ K_1 p_{H_2} \\ DEN = \left(1 + K_2 X_\mathrm{A} + K_3 X_\mathrm{B} + \frac{X_\mathrm{C}}{K_7}\right)\left(1 + K_1 p_{H_2}\right) \end{cases}$$ *Equation 11*

14 parameters must be regressed for Scheme 1(d):

- A_n, $E_{a,n}$ with $n = 4, 5, 6$
- B_m, ΔH_m with $n = 1, 2, 3, 7$

WILEY ◆ VCH

Scheme 1(e)

Elementary steps	Basic routes N(1)	N(2)	N(3)
1) $Z + H_2 \overset{K_1}{\leftrightarrow} ZH_2$	1	1	1
2) $Z + A \overset{K_2}{\leftrightarrow} ZA$	1	0	0
3) $Z + B \overset{K_3}{\leftrightarrow} ZB$	-1	1	0
4) $ZA + ZH_2 \overset{k_4}{\rightarrow} ZB + Z$	1	0	0
5) $ZB + ZH_2 \overset{k_5}{\rightarrow} ZC + Z$	0	1	0
6) $ZC + ZH_2 \overset{k_6}{\rightarrow} D + 2Z$	0	0	1
7) $ZC \overset{K_7}{\leftrightarrow} Z + C$	0	1	-1
	$A \xrightarrow{+H_2} B$	$B \xrightarrow{+H_2} C$	$C \xrightarrow{+H_2} D$

Rates of reactions $r_A,\ r_B,\ r_C,\ r_D$ are related to rates of irreversible surface irreversible reactions (elementary steps) as detailed in Equation S. 28.

$$\begin{cases} r_A = -r_4 \\ r_B = r_4 - r_5 \\ r_C = r_5 - r_6 \\ r_D = r_6 \end{cases} \qquad \textit{Equation S. 28}$$

Rates of irreversible surface irreversible reactions (elementary steps) are in Equation S. 29.

$$\begin{cases} r_4 = k_4\theta_A\theta_{H_2} \\ r_5 = k_5\theta_B\theta_{H_2} \\ r_6 = k_6\theta_C\theta_{H_2} \end{cases} \qquad \textit{Equation S. 29}$$

Equilibrium constants of adsorption/desorption steps are in Equation S. 30, from which expressions in Equation S. 31 can be obtained.

$$\begin{cases} K_1 = \dfrac{\theta_{H_2}}{p_{H_2}\theta_0} \\ K_2 = \dfrac{\theta_A}{X_A\ \theta_0} \\ K_3 = \dfrac{\theta_B}{X_B\ \theta_0} \\ K_7 = \dfrac{X_C\ \theta_0}{\theta_C} \end{cases} \qquad \textit{Equation S. 30}$$

$$\begin{cases} \theta_{H_2} = K_1\theta_0 p_{H_2} \\ \theta_A = K_2\theta_0 X_A \\ \theta_B = K_3\theta_0 X_B \\ \theta_C = \dfrac{X_C\ \theta_0}{K_7} \end{cases} \qquad \textit{Equation S. 31}$$

The balance on sites Z is in Equation S. 32.

$$1 = \theta_0 + \theta_A + \theta_B + \theta_C + \theta_{H_2} \qquad \textit{Equation S. 32}$$

By substituting Equation S. 31 in Equation S. 32, one obtains the expression of θ_0 in Equation S. 33.

WILEY - VCH

$$\theta_0 = \frac{1}{1 + K_2 X_\mathrm{A} + K_3 X_\mathrm{B} + \frac{X_\mathrm{C}}{K_7} + K_1 p_{H_2}}$$ *Equation S. 33*

By substituting Equation S. 31, Equation S. 33, and Equation S. 29 in Equation S. 28, the expression of reaction rates are obtained as functions of observable X_i for Scheme 1(e) (Equation 12).

$$\begin{cases} r_\mathrm{A} = -\frac{k_4\ K_2\ X_\mathrm{A}}{DEN}\ K_1 p_{H_2} \\ r_\mathrm{B} = \frac{k_4\ K_2\ X_\mathrm{A}\ - k_5\ K_3\ X_\mathrm{B}}{DEN}\ K_1 p_{H_2} \\ r_\mathrm{C} = \frac{k_5\ K_3\ X_\mathrm{B}\ - \left(\frac{k_6}{K_7}\right) X_\mathrm{C}}{DEN}\ K_1 p_{H_2} \\ r_\mathrm{D} = \frac{\left(\frac{k_6}{K_7}\right) X_\mathrm{C}}{DEN}\ K_1 p_{H_2} \\ DEN = \left(1 + K_2 X_\mathrm{A} + K_3 X_\mathrm{B} + \frac{X_\mathrm{C}}{K_7} + K_1 p_{H_2}\right)^2 \end{cases}$$ *Equation 12*

14 parameters must be regressed for Scheme 1(e):

- A_n, $E_{a,n}$ with $n\ =\ 4, 5, 6$
- B_m, ΔH_m with $n\ =\ 1, 2, 3, 7$

WILEY ■ VCH

Scheme 1(f)

Elementary steps	Basic routes					
	N(1)	N(2)	N(3)	N(4)	N(5)	N(6)
1) $Z' + H_2 \overset{K_1}{\leftrightarrow} Z'H_2$	1	1	1	0	0	0
2) $Z + H_2 \overset{K_2}{\leftrightarrow} ZH_2$	0	0	0	1	1	1
3) $Z + A \overset{K_3}{\leftrightarrow} ZA$	1	0	0	1	0	0
4) $Z + B \overset{K_4}{\leftrightarrow} ZB$	-1	1	0	-1	1	0
5) $ZA + Z'H_2 \overset{k_5}{\rightarrow} ZB + Z'$	1	0	0	0	0	0
6) $ZA + ZH_2 \overset{k_6}{\rightarrow} ZB + Z$	0	0	0	1	0	0
7) $ZB + Z'H_2 \overset{k_7}{\rightarrow} ZC + Z'$	0	1	0	0	0	0
8) $ZB + ZH_2 \overset{k_8}{\rightarrow} ZC + Z$	0	0	0	0	1	0
9) $ZC + Z'H_2 \overset{k_9}{\rightarrow} D + Z + Z'$	0	0	1	0	0	0
10) $ZC + ZH_2 \overset{k_{10}}{\longrightarrow} D + 2Z$	0	0	0	0	0	1
11) $ZC \overset{K_{11}}{\leftrightarrow} Z + C$	0	1	-1	0	1	-1
	$A \overset{+H_2}{\longrightarrow} B$	$A \overset{+H_2}{\longrightarrow} B$	$B \overset{+H_2}{\longrightarrow} C$	$B \overset{+H_2}{\longrightarrow} C$	$C \overset{+H_2}{\longrightarrow} D$	$C \overset{+H_2}{\longrightarrow} D$

Rates of reactions $r_A,\ r_B,\ r_C,\ r_D$ are related to rates of irreversible surface irreversible reactions (elementary steps) as detailed in Equation S. 34.

$$\begin{cases} r_{\mathrm{A}} = -r_5 - r_6 \\ r_B = r_5 + r_6 - r_7 - r_8 \\ r_C = r_7 + r_8 - r_9 - r_{10} \\ r_D = r_9 + r_{10} \end{cases}$$ *Equation S. 34*

Rates of irreversible surface irreversible reactions (elementary steps) are in Equation S. 35.

$$\begin{cases} r_5 = k_5\theta_{\mathrm{A}}\theta'_{\mathrm{H_2}} \\ r_6 = k_6\theta_{\mathrm{A}}\theta_{\mathrm{H_2}} \\ r_7 = k_7\theta_{\mathrm{B}}\theta'_{\mathrm{H_2}} \\ r_8 = k_8\theta_{\mathrm{B}}\theta_{\mathrm{H_2}} \\ r_9 = k_9\theta_C\theta'_{\mathrm{H_2}} \\ r_{10} = k_{10}\theta_C\theta_{\mathrm{H_2}} \end{cases}$$ *Equation S. 35*

Equilibrium constants of adsorption/desorption steps are in Equation S. 36, from which expressions in Equation S. 37 can be obtained.

$$\begin{cases} K_1 = \dfrac{\theta'_{\mathrm{H_2}}}{p_{H_2}\left(1 - \theta'_{\mathrm{H_2}}\right)} \\ K_2 = \dfrac{\theta_{\mathrm{H_2}}}{p_{H_2}\theta_0} \\ K_3 = \dfrac{\theta_{\mathrm{A}}}{X_{\mathrm{A}}\ \theta_0} \\ K_4 = \dfrac{\theta_{\mathrm{B}}}{X_{\mathrm{B}}\ \theta_0} \\ K_{11} = \dfrac{X_{\mathrm{C}}\ \theta_0}{\theta_{\mathrm{C}}} \end{cases}$$ *Equation S. 36*

WILEY-VCH

$$\begin{cases} \theta'_{\mathrm{H_2}} = \dfrac{K_1 p_{H_2}}{1 + K_1 p_{H_2}} \\ \theta_{\mathrm{H_2}} = K_2 \theta_0 p_{H_2} \\ \theta_{\mathrm{A}} = K_3 \theta_0 X_{\mathrm{A}} \\ \theta_{\mathrm{B}} = K_4 \theta_0 X_{\mathrm{B}} \\ \theta_{\mathrm{C}} = \dfrac{X_{\mathrm{C}}\, \theta_0}{K_{11}} \end{cases} \quad \text{Equation S. 37}$$

The balance on sites Z is in Equation S. 38. The balance on sites Z′ is already implied in Equation S. 36 ($1 = \theta'_0 + \theta'_{\mathrm{H_2}}$).

$$1 = \theta_0 + \theta_{\mathrm{A}} + \theta_{\mathrm{B}} + \theta_{\mathrm{C}} + \theta_{\mathrm{H_2}} \quad \text{Equation S. 38}$$

By substituting Equation S. 37 in Equation S. 38, one obtains the expression of θ_0 in Equation S. 39.

$$\theta_0 = \frac{1}{1 + K_3 X_{\mathrm{A}} + K_4 X_{\mathrm{B}} + \dfrac{X_{\mathrm{C}}}{K_{11}} + K_2 p_{H_2}} \quad \text{Equation S. 39}$$

By substituting Equation S. 37, Equation S. 39, and Equation S. 35 in Equation S. 34, the expression of reaction rates are obtained as functions of observable X_i for Scheme 1(f) (Equation 13).

$$\begin{cases} r_{\mathrm{A}} = -\left(\dfrac{k_5\, K_3\, X_{\mathrm{A}}}{DEN1}\, K_1 p_{H_2} + \dfrac{k_6\, K_3\, X_{\mathrm{A}}}{DEN2}\, K_2 p_{H_2}\right) \\ r_{\mathrm{B}} = \dfrac{k_5\, K_3\, X_{\mathrm{A}} - k_7\, K_4\, X_{\mathrm{B}}}{DEN1}\, K_1 p_{H_2} + \dfrac{k_6\, K_3\, X_{\mathrm{A}} - k_8\, K_4\, X_{\mathrm{B}}}{DEN2}\, K_2 p_{H_2} \\ r_C = \dfrac{k_7\, K_4\, X_{\mathrm{B}} - \left(\dfrac{k_9}{K_{11}}\right) X_{\mathrm{C}}}{DEN1}\, K_1 p_{H_2} + \dfrac{k_8\, K_4\, X_{\mathrm{B}} - \left(\dfrac{k_{10}}{K_{11}}\right) X_{\mathrm{C}}}{DEN2}\, K_2 p_{H_2} \\ r_D = \dfrac{\left(\dfrac{k_9}{K_{11}}\right) X_{\mathrm{C}}}{DEN1}\, K_1 p_{H_2} + \dfrac{\left(\dfrac{k_{10}}{K_{11}}\right) X_{\mathrm{C}}}{DEN2}\, K_2 p_{H_2} \\ DEN1 = \left(1 + K_3 X_{\mathrm{A}} + K_4 X_{\mathrm{B}} + \dfrac{X_{\mathrm{C}}}{K_{11}} + K_2 p_{H_2}\right)\left(1 + K_1 p_{H_2}\right) \\ DEN2 = \left(1 + K_3 X_{\mathrm{A}} + K_4 X_{\mathrm{B}} + \dfrac{X_{\mathrm{C}}}{K_{11}} + K_2 p_{H_2}\right)^2 \end{cases} \quad \text{Equation 13}$$

22 parameters must be regressed for Scheme 1(f):

- A_n, $E_{a,n}$ with $n = 5, 6, 7, 8, 9, 10$
- B_m, ΔH_m with $n = 1, 2, 3, 4, 11$

WILEY ■ VCH

Main operations in MATLAB® 2023b regression script

1. Start
2. Load experimental datasets for Tests 3, 4, 5 (Table S.1, Table S.2, Table S.3) and related conditions (Table 1)
3. Concatenate data and set indexing arrays
4. Select reaction mechanism (Scheme 1 in the main text)
5. Set initial values of parameters A_n, $E_{a,n}$, B_m, ΔH_m and related bounds, and reactor model ODE (Equation 4 I the main text) with kinetic rate laws according to choice at point 4
6. Run parameter estimation using nonlinear least squares (`lsqnonlin`) applied to the ODE solution (`ode15s`)
7. Compute statistical analysis: covariance matrix of the vector $\boldsymbol{x}$ of estimated parameters (Equation S. 40), standard deviation of each regressed parameter x_n (i.e., Standard Error, SE_n, Equation S. 41), percentage standard deviation (i.e., Relative Standard Error, RSE_n, Equation S. 42), together with SSR_{global} (Equation 5), R^2 (Equation 6), and $SSR_{i,h}$ (Equation 7) defined in the main text.

$$\boldsymbol{Var}(\boldsymbol{x}) = \sigma^2 \left(\boldsymbol{J}^T\boldsymbol{J}\right)^{-1} \quad \text{Equation S. 40}$$

$$SE_n = std(x_n) = \sqrt{|\boldsymbol{Var}(\boldsymbol{x})|_{\boldsymbol{nn}}} \quad \text{Equation S. 41}$$

$$RSE_n = \left(\frac{std(x_n)}{x_n}\right) \times 100 \quad \text{Equation S. 42}$$

where:

- $\boldsymbol{J}$ is the Jacobian matrix evaluated at the solution.
- $\sigma^2 = \frac{SSR_{global}}{N-p}$ is the estimated variance of the residuals.
- N is the total number of experimental data points.
- p is the number of parameters estimated ($n = 1, \dots, p$).

8. Display estimated parameters and statistical metrics

WILEY-VCH

Regressed parameters

Table S.4. Kinetic parameters regressed for Equation 8, i.e., for rate laws of HCSH of VO by Lindlar catalyst interpreted by the reaction mechanism in Scheme 1(a). Note: “E±XX” = “·$10^{\pm XX}$”

Parameter	Units	*Value* x_n	SE_n	RSE_n (%)
B_1	bar_{H2}^{-1}	3.53E-06	2.06E-06	58.4
B_2	$mol_{tot}\ mol_{C18:3}^{-1}$	2.66E+00	9.95E-01	37.4
B_3	$mol_{tot}\ mol_{C18:2}^{-1}$	3.59E+00	7.90E-01	22.0
B_{10}	$mol_{C18:1}\ mol_{tot}^{-1}$	1.96E-01	6.51E-02	33.2
ΔH_1	J mol^{-1}	-1.54E+04	3.30E-05	0.0
ΔH_2	J mol^{-1}	-7.79E+03	6.26E-06	0.0
ΔH_3	J mol^{-1}	-8.44E+01	9.08E-04	0.0
ΔH_{10}	J mol^{-1}	-3.01E+03	1.14E-05	0.0
A_4	$mol_{C18:3}\ mol_{tot}^{-1}\ min^{-1}$	1.60E+01	1.64E-01	1.0
A_5	$mol_{C18:3}\ mol_{tot}^{-1}\ min^{-1}$	7.02E-01	5.52E-01	78.7
A_6	$mol_{C18:2}\ mol_{tot}^{-1}\ min^{-1}$	1.37E+01	2.05E-01	1.5
A_7	$mol_{C18:2}\ mol_{tot}^{-1}\ min^{-1}$	2.32E+00	1.56E-01	6.8
A_8	$mol_{C18:1}\ mol_{tot}^{-1}\ min^{-1}$	1.24E+01	6.60E-03	0.0
A_9	$mol_{C18:1}\ mol_{tot}^{-1}\ min^{-1}$	8.66E-01	2.81E-01	32.5
$E_{a,4}$	J mol^{-1}	7.36E+04	1.61E-06	0.0
$E_{a,5}$	J mol^{-1}	1.40E+04	1.32E-05	0.0
$E_{a,6}$	J mol^{-1}	7.08E+04	1.49E-05	0.0
$E_{a,7}$	J mol^{-1}	1.50E+04	1.07E-05	0.0
$E_{a,8}$	J mol^{-1}	6.00E+04	2.15E-05	0.0
$E_{a,9}$	J mol^{-1}	1.20E+03	4.55E-05	0.0

WILEY-VCH

Table S.5. Covariance matrix of kinetic parameters regressed for Equation 8, i.e., for rate laws of HCSH of VO by Lindlar catalyst interpreted by the reaction mechanism in Scheme 1(a). Note: “E±XX” = “·10^{±XX}”

	B_1	B_2	B_3	B_{10}	ΔH_1	ΔH_2	ΔH_3	ΔH_{10}	A_4	A_5	A_6	A_7	A_8	A_9	$E_{a,4}$	$E_{a,5}$	$E_{a,6}$	$E_{a,7}$	$E_{a,8}$	$E_{a,9}$
B_1	4.24E-12	8.04E-07	-2.99E-07	-1.05E-07	-6.52E-11	8.01E-12	5.01E-10	-2.16E-11	1.36E-07	-9.03E-07	-6.35E-08	-2.10E-07	1.21E-08	-5.25E-07	2.15E-13	-2.17E-11	-2.86E-11	-1.99E-11	-3.66E-11	-2.19E-11
B_2	8.04E-07	9.89E-01	-7.64E-01	-3.08E-02	-1.30E-05	5.95E-06	8.90E-04	-7.72E-06	1.63E-01	-3.10E-02	-1.96E-01	3.88E-02	1.13E-03	-1.24E-01	1.51E-06	1.39E-06	-6.14E-06	-1.86E-06	-1.77E-05	3.49E-05
B_3	-2.99E-07	-7.64E-01	6.24E-01	1.71E-02	4.46E-06	-4.26E-06	-7.14E-04	4.56E-06	-1.26E-01	-7.69E-02	1.62E-01	-5.77E-02	3.21E-04	6.30E-02	-1.26E-06	-3.51E-06	2.22E-06	-5.08E-07	1.16E-05	-3.23E-05
B_{10}	-1.05E-07	-3.08E-02	1.71E-02	4.24E-03	1.50E-06	-2.50E-07	-2.29E-05	5.30E-07	-5.16E-03	1.94E-02	4.14E-03	3.87E-03	-2.34E-04	1.15E-02	-2.71E-08	4.22E-07	6.75E-07	4.31E-07	9.78E-07	-6.21E-08
ΔH_1	-6.52E-11	-1.30E-05	4.46E-06	1.50E-06	1.09E-09	-1.33E-10	-7.48E-09	3.42E-10	-2.21E-06	1.64E-05	9.26E-07	3.88E-06	-2.10E-07	7.64E-06	-3.97E-12	3.77E-10	4.90E-10	3.41E-10	5.87E-10	4.12E-10
ΔH_2	8.01E-12	5.95E-06	-4.26E-06	-2.50E-07	-1.33E-10	3.92E-11	5.09E-09	-6.03E-11	9.86E-07	-1.18E-06	-1.08E-06	-3.86E-08	1.86E-08	-1.07E-06	8.16E-12	-1.57E-11	-6.18E-11	-3.04E-11	-1.27E-10	1.57E-10
ΔH_3	5.01E-10	8.90E-04	-7.14E-04	-2.29E-05	-7.48E-09	5.09E-09	8.25E-07	-6.01E-09	1.47E-04	5.59E-05	-1.84E-04	5.79E-05	8.77E-08	-9.41E-05	1.42E-09	3.17E-09	-3.54E-09	-1.57E-10	-1.45E-08	3.56E-08
ΔH_{10}	-2.16E-11	-7.72E-06	4.56E-06	5.30E-07	3.42E-10	-6.03E-11	-6.01E-09	1.30E-10	-1.29E-06	4.02E-06	1.11E-06	7.37E-07	-5.86E-08	2.93E-06	-7.25E-12	8.86E-11	1.51E-10	9.49E-11	2.38E-10	-5.31E-11
A_4	1.36E-07	1.63E-01	-1.26E-01	-5.16E-03	-2.21E-06	9.86E-07	1.47E-04	-1.29E-06	2.69E-02	-6.19E-03	-3.22E-02	6.11E-03	2.00E-04	-2.09E-02	2.48E-07	2.02E-07	-1.04E-06	-3.29E-07	-2.95E-06	5.69E-06
A_5	-9.03E-07	-3.10E-02	-7.69E-02	1.94E-02	1.64E-05	-1.18E-06	5.59E-05	4.02E-06	-6.19E-03	3.05E-01	-2.34E-02	8.23E-02	-3.52E-03	8.27E-02	2.03E-07	7.10E-06	7.59E-06	5.74E-06	6.26E-06	1.46E-05
A_6	-6.35E-08	-1.96E-01	1.62E-01	4.14E-03	9.26E-07	-1.08E-06	-1.84E-04	1.11E-06	-3.22E-02	-2.34E-02	4.19E-02	-1.58E-02	1.29E-04	1.46E-02	-3.27E-07	-9.94E-07	4.73E-07	-2.05E-07	2.90E-06	-8.50E-06
A_7	-2.10E-07	3.88E-02	-5.77E-02	3.87E-03	3.88E-06	-3.86E-08	5.79E-05	7.37E-07	6.11E-03	8.23E-02	-1.58E-02	2.45E-02	-9.13E-04	1.70E-02	1.28E-07	2.02E-06	1.79E-06	1.49E-06	8.75E-07	5.69E-06
A_8	1.21E-08	1.13E-03	3.21E-04	-2.34E-04	-2.10E-07	1.86E-08	8.77E-08	-5.86E-08	2.00E-04	-3.52E-03	1.29E-04	-9.13E-04	4.36E-05	-1.33E-03	-1.54E-09	-8.33E-08	-9.49E-08	-7.00E-08	-9.40E-08	-1.47E-07
A_9	-5.25E-07	-1.24E-01	6.30E-02	1.15E-02	7.64E-06	-1.07E-06	-9.41E-05	2.93E-06	-2.09E-02	8.27E-02	1.46E-02	1.70E-02	-1.33E-03	7.91E-02	-6.16E-08	2.15E-06	3.17E-06	2.14E-06	4.95E-06	7.67E-07
$E_{a,4}$	2.15E-13	1.51E-06	-1.26E-06	-2.71E-08	-3.97E-12	8.16E-12	1.42E-09	-7.25E-12	2.48E-07	2.03E-07	-3.27E-07	1.28E-07	-1.54E-09	-6.16E-08	2.60E-12	8.68E-12	-2.58E-12	2.46E-12	-2.06E-11	6.76E-11
$E_{a,5}$	-2.17E-11	1.39E-06	-3.51E-06	4.22E-07	3.77E-10	-1.57E-11	3.17E-09	8.86E-11	2.02E-07	7.10E-06	-9.94E-07	2.02E-06	-8.33E-08	2.15E-06	8.68E-12	1.75E-10	1.70E-10	1.35E-10	1.23E-10	4.29E-10
$E_{a,6}$	-2.86E-11	-6.14E-06	2.22E-06	6.75E-07	4.90E-10	-6.18E-11	-3.54E-09	1.51E-10	-1.04E-06	7.59E-06	4.73E-07	1.79E-06	-9.49E-08	3.17E-06	-2.58E-12	1.70E-10	2.23E-10	1.55E-10	2.63E-10	1.78E-10
$E_{a,7}$	-1.99E-11	-1.86E-06	-5.08E-07	4.31E-07	3.41E-10	-3.04E-11	-1.57E-10	9.49E-11	-3.29E-07	5.74E-06	-2.05E-07	1.49E-06	-7.00E-08	2.14E-06	2.46E-12	1.35E-10	1.55E-10	1.14E-10	1.53E-10	2.38E-10
$E_{a,8}$	-3.66E-11	-1.77E-05	1.16E-05	9.78E-07	5.87E-10	-1.27E-10	-1.45E-08	2.38E-10	-2.95E-06	6.26E-06	2.90E-06	8.75E-07	-9.40E-08	4.95E-06	-2.06E-11	1.23E-10	2.63E-10	1.53E-10	4.62E-10	-3.00E-10
$E_{a,9}$	-2.19E-11	3.49E-05	-3.23E-05	-6.21E-08	4.12E-10	1.57E-10	3.56E-08	-5.31E-11	5.69E-06	1.46E-05	-8.50E-06	5.69E-06	-1.47E-07	7.67E-07	6.76E-11	4.29E-10	1.78E-10	2.38E-10	-3.00E-10	2.07E-09

WILEY-VCH

Table S.6. Kinetic parameters regressed for Equation 9, i.e., for rate laws of HCSH of VO by Lindlar catalyst interpreted by the reaction mechanism in Scheme 1(b). Note: "E±XX" = "$\cdot 10^{\pm XX}$"

Parameter	Units	***Value*** x_n	SE_n	RSE_n (%)
B_1	bar_{H2}^{-1}	3.76E-06	1.61E-06	42.9
B_2	$mol_{tot}\ mol_{C18:3}^{-1}$	1.64E+00	8.80E-01	53.7
B_3	$mol_{tot}\ mol_{C18:2}^{-1}$	3.38E+00	1.80E+00	53.2
B_{10}	$mol_{C18:1}\ mol_{tot}^{-1}$	1.54E-01	5.58E-02	36.3
ΔH_1	J mol^{-1}	-5.42E+03	2.03E-04	0.0
ΔH_2	J mol^{-1}	-8.73E+03	1.81E-05	0.0
ΔH_3	J mol^{-1}	-2.59E+03	5.53E-05	0.0
ΔH_{10}	J mol^{-1}	-1.74E+02	1.65E-04	0.0
A_4	$mol_{C18:3}\ mol_{tot}^{-1}\ min^{-1}$	7.49E+00	2.29E-01	3.1
A_5	$mol_{C18:3}\ mol_{tot}^{-1}\ min^{-1}$	1.00E+01	6.21E-02	0.0
A_6	$mol_{C18:2}\ mol_{tot}^{-1}\ min^{-1}$	1.02E+01	3.74E-01	3.7
A_7	$mol_{C18:2}\ mol_{tot}^{-1}\ min^{-1}$	9.22E+00	3.09E-01	3.4
A_8	$mol_{C18:1}\ mol_{tot}^{-1}\ min^{-1}$	1.14E+01	2.66E-02	0.0
A_9	$mol_{C18:1}\ mol_{tot}^{-1}\ min^{-1}$	2.83E+00	1.09E+00	38.4
$E_{a,4}$	J mol^{-1}	3.44E+04	1.86E-05	0.0
$E_{a,5}$	J mol^{-1}	5.41E+03	1.03E-05	0.0
$E_{a,6}$	J mol^{-1}	4.82E+04	6.25E-05	0.0
$E_{a,7}$	J mol^{-1}	4.95E+03	3.57E-05	0.0
$E_{a,8}$	J mol^{-1}	3.42E+04	2.02E-04	0.0
$E_{a,9}$	J mol^{-1}	8.05E+03	1.39E-04	0.0

WILEY-VCH

WILEY-VCH

Table S.7. Covariance matrix of kinetic parameters regressed for Equation 9, i.e., for rate laws of HCSH of VO by Lindlar catalyst interpreted by the reaction mechanism in Scheme 1(b). Note: "E±XX" = "·$10^{±XX}$"

	B_1	B_2	B_3	B_{10}	ΔH_1	ΔH_2	ΔH_3	ΔH_{10}	A_4	A_5	A_6	A_7	A_8	A_9	$E_{a,4}$	$E_{a,5}$	$E_{a,6}$	$E_{a,7}$	$E_{a,8}$	$E_{a,9}$
B_1	2.60E-12	3.37E-07	-1.26E-06	-5.28E-08	-3.16E-10	1.67E-11	5.83E-11	-2.06E-10	2.42E-07	3.49E-08	3.60E-08	1.86E-07	4.05E-08	-1.69E-06	-2.25E-11	-1.41E-11	-6.67E-11	-3.81E-11	-2.78E-10	-2.18E-10
B_2	3.37E-07	7.74E-01	-2.89E-02	6.68E-04	-2.82E-05	2.29E-06	3.31E-06	-2.96E-06	1.61E-01	-1.24E-02	-1.89E-02	-6.66E-02	6.08E-03	-1.17E-01	-1.98E-06	-2.70E-06	-9.41E-06	-1.13E-05	-1.20E-05	-1.95E-05
B_3	-1.26E-06	-2.89E-02	3.22E+00	6.87E-02	1.92E-04	1.48E-05	-9.56E-05	2.62E-04	-2.24E-01	-1.08E-01	5.71E-01	-5.36E-01	-3.04E-02	8.62E-01	3.05E-05	-9.95E-07	-3.92E-05	-2.20E-05	2.95E-04	1.25E-04
B_{10}	-5.28E-08	6.68E-04	6.87E-02	3.12E-03	6.31E-06	1.10E-07	-2.23E-06	6.60E-06	-5.37E-03	-2.31E-03	9.56E-03	-1.16E-02	-8.76E-04	3.10E-02	7.45E-07	1.05E-07	-9.03E-08	-7.90E-08	7.82E-06	4.20E-06
ΔH_1	-3.16E-10	-2.82E-05	1.92E-04	6.31E-06	4.13E-08	-1.90E-09	-8.28E-09	2.87E-08	-2.98E-05	-5.78E-06	1.34E-06	-3.04E-05	-5.31E-06	2.19E-04	3.12E-09	1.71E-09	7.76E-09	4.34E-09	3.79E-08	2.83E-08
ΔH_2	1.67E-11	2.29E-06	1.48E-05	1.10E-07	-1.90E-09	3.28E-10	-1.99E-10	-1.36E-11	4.96E-07	-5.59E-07	5.69E-06	-2.61E-06	1.86E-07	-1.17E-05	1.95E-11	-1.70E-10	-1.12E-09	-6.29E-10	-5.42E-10	-1.38E-09
ΔH_3	5.83E-11	3.31E-06	-9.56E-05	-2.23E-06	-8.28E-09	-1.99E-10	3.06E-09	-8.90E-09	8.10E-06	3.15E-06	-1.41E-05	1.57E-05	1.21E-06	-4.01E-05	-1.02E-09	-1.24E-10	2.87E-10	1.61E-10	-1.05E-08	-5.50E-09
ΔH_{10}	-2.06E-10	-2.96E-06	2.62E-04	6.60E-06	2.87E-08	-1.36E-11	-8.90E-09	2.72E-08	-2.33E-05	-8.68E-06	3.15E-05	-4.39E-05	-3.96E-06	1.44E-04	3.04E-09	6.77E-10	1.23E-09	5.93E-10	3.30E-08	1.93E-08
A_4	2.42E-07	1.61E-01	-2.24E-01	-5.37E-03	-2.98E-05	4.96E-07	8.10E-06	-2.33E-05	5.26E-02	4.68E-03	-2.99E-02	2.29E-02	4.57E-03	-1.45E-01	-2.95E-06	-1.13E-06	-3.01E-06	-2.84E-06	-3.00E-05	-2.02E-05
A_5	3.49E-08	-1.24E-02	-1.08E-01	-2.31E-03	-5.78E-06	-5.59E-07	3.15E-06	-8.68E-06	4.68E-03	3.86E-03	-1.92E-02	1.92E-02	8.96E-04	-2.58E-02	-9.86E-07	9.28E-08	1.56E-06	9.75E-07	-9.58E-06	-3.71E-06
A_6	3.60E-08	-1.89E-02	5.71E-01	9.56E-03	1.34E-06	5.69E-06	-1.41E-05	3.15E-05	-2.99E-02	-1.92E-02	1.40E-01	-9.28E-02	-1.70E-03	-3.43E-02	4.02E-06	-2.06E-06	-1.80E-05	-9.74E-06	2.94E-05	-8.50E-07
A_7	1.86E-07	-6.66E-02	-5.36E-01	-1.16E-02	-3.04E-05	-2.61E-06	1.57E-05	-4.39E-05	2.29E-02	1.92E-02	-9.28E-02	9.57E-02	4.62E-03	-1.38E-01	-4.96E-06	3.68E-07	7.14E-06	4.56E-06	-4.87E-05	-1.96E-05
A_8	4.05E-08	6.08E-03	-3.04E-02	-8.76E-04	-5.31E-06	1.86E-07	1.21E-06	-3.96E-06	4.57E-03	8.96E-04	-1.70E-03	4.62E-03	7.07E-04	-2.77E-02	-4.42E-07	-2.02E-07	-8.19E-07	-4.79E-07	-5.13E-06	-3.62E-06
A_9	-1.69E-06	-1.17E-01	8.62E-01	3.10E-02	2.19E-04	-1.17E-05	-4.01E-05	1.44E-04	-1.45E-01	-2.58E-02	-3.43E-02	-1.38E-01	-2.77E-02	1.18E+00	1.54E-05	9.65E-06	4.65E-05	2.56E-05	1.94E-04	1.51E-04
$E_{a,4}$	-2.25E-11	-1.98E-06	3.05E-05	7.45E-07	3.12E-09	1.95E-11	-1.02E-09	3.04E-09	-2.95E-06	-9.86E-07	4.02E-06	-4.96E-06	-4.42E-07	1.54E-05	3.46E-10	6.83E-11	6.87E-11	4.23E-11	3.67E-09	2.09E-09
$E_{a,5}$	-1.41E-11	-2.70E-06	-9.95E-07	1.05E-07	1.71E-09	-1.70E-10	-1.24E-10	6.77E-10	-1.13E-06	9.28E-08	-2.06E-06	3.68E-07	-2.02E-07	9.65E-06	6.83E-11	1.07E-10	6.14E-10	3.52E-10	1.10E-09	1.20E-09
$E_{a,6}$	-6.67E-11	-9.41E-06	-3.92E-05	-9.03E-08	7.76E-09	-1.12E-09	2.87E-10	1.23E-09	-3.01E-06	1.56E-06	-1.80E-05	7.14E-06	-8.19E-07	4.65E-05	6.87E-11	6.14E-10	3.91E-09	2.19E-09	3.30E-09	5.56E-09
$E_{a,7}$	-3.81E-11	-1.13E-05	-2.20E-05	-7.90E-08	4.34E-09	-6.29E-10	1.61E-10	5.93E-10	-2.84E-06	9.75E-07	-9.74E-06	4.56E-06	-4.79E-07	2.56E-05	4.23E-11	3.52E-10	2.19E-09	1.28E-09	1.77E-09	3.10E-09
$E_{a,8}$	-2.78E-10	-1.20E-05	2.95E-04	7.82E-06	3.79E-08	-5.42E-10	-1.05E-08	3.30E-08	-3.00E-05	-9.58E-06	2.94E-05	-4.87E-05	-5.13E-06	1.94E-04	3.67E-09	1.10E-09	3.30E-09	1.77E-09	4.09E-08	2.56E-08
$E_{a,9}$	-2.18E-10	-1.95E-05	1.25E-04	4.20E-06	2.83E-08	-1.38E-09	-5.50E-09	1.93E-08	-2.02E-05	-3.71E-06	-8.50E-07	-1.96E-05	-3.62E-06	1.51E-04	2.09E-09	1.20E-09	5.56E-09	3.10E-09	2.56E-08	1.94E-08

Table S.8. Kinetic parameters regressed for Equation 10, i.e., for rate laws of HCSH of VO by Lindlar catalyst interpreted by the reaction mechanism in Scheme 1(c). Note: "E±XX" = "$\cdot 10^{\pm XX}$"

Parameter	Units	*Value* x_n	SE_n	RSE_n (%)
B_1	$mol_{tot}\ mol_{C18:3}^{-1}$	6.94E+00	2.99E+01	431.5
B_2	$mol_{tot}\ mol_{C18:2}^{-1}$	2.33E+00	5.53E+00	237.8
B_9	$mol_{C18:1}\ mol_{tot}^{-1}$	6.65E-03	1.80E-01	2706.7
ΔH_1	J mol^{-1}	-3.82E+04	2.05E+00	0.0
ΔH_2	J mol^{-1}	-1.06E+03	2.27E+02	-21.4
ΔH_9	J mol^{-1}	-4.09E+04	3.47E+02	-0.8
A_3	$mol_{C18:3}\ mol_{tot}^{-1}\ min^{-1}\ bar_{H2}^{-1}$	2.19E-03	9.34E-03	426.8
A_4	$mol_{C18:3}\ mol_{tot}^{-1}\ min^{-1}$	3.65E+01	3.86E+02	1057.6
A_5	$mol_{C18:2}\ mol_{tot}^{-1}\ min^{-1}\ bar_{H2}^{-1}$	2.33E-03	5.60E-03	240.3
A_6	$mol_{C18:2}\ mol_{tot}^{-1}\ min^{-1}$	3.01E-02	4.31E-02	143.3
A_7	$mol_{C18:1}\ mol_{tot}^{-1}\ min^{-1}\ bar_{H2}^{-1}$	6.22E-02	1.68E+00	2700.5
A_8	$mol_{C18:1}\ mol_{tot}^{-1}\ min^{-1}$	9.96E+00	2.08E+02	2092.7
$E_{a,3}$	J mol^{-1}	8.92E+00	2.23E+02	2502.4
$E_{a,4}$	J mol^{-1}	3.35E+00	3.53E+03	105564.9
$E_{a,5}$	J mol^{-1}	3.16E+04	1.99E+02	0.6
$E_{a,6}$	J mol^{-1}	3.48E+03	1.08E+01	0.3
$E_{a,7}$	J mol^{-1}	6.44E+04	3.48E+02	0.5
$E_{a,8}$	J mol^{-1}	7.40E+04	1.81E-01	0.0

WILEY-VCH

Table S.9. Covariance matrix of kinetic parameters regressed for Equation 10, i.e., for rate laws of HCSH of VO by Lindlar catalyst interpreted by the reaction mechanism in Scheme 1(c). Note: "E±XX" = "·$10^{\pm XX}$"

	B_1	B_2	B_9	ΔH_1	ΔH_2	ΔH_9	A_3	A_4	A_5	A_6	A_7	A_8	$E_{a,3}$	$E_{a,4}$	$E_{a,5}$	$E_{a,6}$	$E_{a,7}$	$E_{a,8}$
B_1	8.96E+02	-1.36E+02	4.26E+00	6.65E-01	1.96E+02	-3.41E+02	-2.76E-01	7.88E+02	1.43E-01	2.68E-01	-3.97E+01	1.43E+03	-2.15E+03	3.54E+03	1.95E+02	3.79E-01	-3.40E+02	-4.65E-02
B_2	-1.36E+02	3.06E+01	-8.84E-01	1.08E+00	9.53E+01	-1.38E+02	4.30E-02	-3.87E+02	-3.07E-02	2.93E-02	8.25E+00	-2.04E+02	2.89E+02	1.39E+03	7.92E+01	-6.50E+00	-1.39E+02	-1.03E-01
B_9	4.26E+00	-8.84E-01	3.24E-02	-7.39E-03	-1.00E-01	-6.81E-02	-1.34E-03	7.08E+00	9.14E-04	-8.67E-04	-3.02E-01	9.65E+00	-8.01E+00	1.19E+00	3.91E-02	6.62E-02	-5.84E-02	1.03E-03
ΔH_1	6.65E-01	1.08E+00	-7.39E-03	4.21E+00	4.65E+02	-7.11E+02	3.37E-05	-7.87E+02	-9.13E-04	2.82E-02	6.80E-02	4.09E+02	-2.14E+02	7.24E+03	4.08E+02	-2.21E+01	-7.14E+02	-3.70E-01
ΔH_2	1.96E+02	9.53E+01	-1.00E-01	4.65E+02	5.15E+04	-7.88E+04	-3.47E-02	-8.68E+04	-7.59E-02	3.11E+00	8.27E-01	4.54E+04	-2.49E+04	8.02E+05	4.52E+04	-2.44E+03	-7.91E+04	-4.10E+01
ΔH_9	-3.41E+02	-1.38E+02	-6.81E-02	-7.11E+02	-7.88E+04	1.20E+05	6.59E-02	1.33E+05	1.08E-01	-4.76E+00	8.04E-01	-6.95E+04	3.86E+04	-1.23E+06	-6.91E+04	3.73E+03	1.21E+05	6.26E+01
A_3	-2.76E-01	4.30E-02	-1.34E-03	3.37E-05	-3.47E-02	6.59E-02	8.72E-05	-2.93E-01	-4.53E-05	-8.38E-05	1.25E-02	-4.27E-01	6.56E-01	-6.92E-01	-3.76E-02	-1.39E-03	6.56E-02	-7.08E-06
A_4	7.88E+02	-3.87E+02	7.08E+00	-7.87E+02	-8.68E+04	1.33E+05	-2.93E-01	1.49E+05	3.61E-01	-5.36E+00	-6.59E+01	-7.48E+04	3.35E+04	-1.35E+06	-7.61E+04	4.14E+03	1.33E+05	6.91E+01
A_5	1.43E-01	-3.07E-02	9.14E-04	-9.13E-04	-7.59E-02	1.08E-01	-4.53E-05	3.61E-01	3.14E-05	-3.04E-05	-8.54E-03	2.24E-01	-3.23E-01	-1.09E+00	-6.21E-02	5.64E-03	1.09E-01	8.74E-05
A_6	2.68E-01	2.93E-02	-8.67E-04	2.82E-02	3.11E+00	-4.76E+00	-8.38E-05	-5.36E+00	-3.04E-05	1.86E-03	7.99E-03	2.43E+00	-1.73E+00	4.84E+01	2.73E+00	-1.49E-01	-4.77E+00	-2.51E-03
A_7	-3.97E+01	8.25E+00	-3.02E-01	6.80E-02	8.27E-01	8.04E-01	1.25E-02	-6.59E+01	-8.54E-03	7.99E-03	2.82E+00	-9.02E+01	7.48E+01	-1.28E+01	-4.62E-01	-6.13E-01	7.15E-01	-9.55E-03
A_8	1.43E+03	-2.04E+02	9.65E+00	4.09E+02	4.54E+04	-6.95E+04	-4.27E-01	-7.48E+04	2.24E-01	2.43E+00	-9.02E+01	4.34E+04	-2.21E+04	7.08E+05	3.99E+04	-2.14E+03	-6.98E+04	-3.58E+01
$E_{a,3}$	-2.15E+03	2.89E+02	-8.01E+00	-2.14E+02	-2.49E+04	3.86E+04	6.56E-01	3.35E+04	-3.23E-01	-1.73E+00	7.48E+01	-2.21E+04	4.98E+04	-3.93E+05	-2.21E+04	1.11E+03	3.87E+04	2.03E+01
$E_{a,4}$	3.54E+03	1.39E+03	1.19E+00	7.24E+03	8.02E+05	-1.23E+06	-6.92E-01	-1.35E+06	-1.09E+00	4.84E+01	-1.28E+01	7.08E+05	-3.93E+05	1.25E+07	7.04E+05	-3.80E+04	-1.23E+06	-6.38E+02
$E_{a,5}$	1.95E+02	7.92E+01	3.91E-02	4.08E+02	4.52E+04	-6.91E+04	-3.76E-02	-7.61E+04	-6.21E-02	2.73E+00	-4.62E-01	3.99E+04	-2.21E+04	7.04E+05	3.97E+04	-2.14E+03	-6.94E+04	-3.60E+01
$E_{a,6}$	3.79E-01	-6.50E+00	6.62E-02	-2.21E+01	-2.44E+03	3.73E+03	-1.39E-03	4.14E+03	5.64E-03	-1.49E-01	-6.13E-01	-2.14E+03	1.11E+03	-3.80E+04	-2.14E+03	1.16E+02	3.75E+03	1.94E+00
$E_{a,7}$	-3.40E+02	-1.39E+02	-5.84E-02	-7.14E+02	-7.91E+04	1.21E+05	6.56E-02	1.33E+05	1.09E-01	-4.77E+00	7.15E-01	-6.98E+04	3.87E+04	-1.23E+06	-6.94E+04	3.75E+03	1.21E+05	6.29E+01
$E_{a,8}$	-4.65E-02	-1.03E-01	1.03E-03	-3.70E-01	-4.10E+01	6.26E+01	-7.08E-06	6.91E+01	8.74E-05	-2.51E-03	-9.55E-03	-3.58E+01	2.03E+01	-6.38E+02	-3.60E+01	1.94E+00	6.29E+01	3.26E-02

WILEY-VCH

Table S.10. Kinetic parameters regressed for Equation 11, i.e., for rate laws of HCSH of VO by Lindlar catalyst interpreted by the reaction mechanism in Scheme 1(d). Note: "E±XX" = "$\cdot 10^{\pm XX}$"

Parameter	Units	***Value*** x_n	SE_n	RSE_n **(%)**
B_1	bar_{H2}^{-1}	1.16E-01	8.66E-03	7.5
B_2	$mol_{tot}\ mol_{C18:3}^{-1}$	1.12E+01	6.52E+00	58.4
B_3	$mol_{tot}\ mol_{C18:2}^{-1}$	7.13E+01	1.77E+01	24.9
B_7	$mol_{C18:1}\ mol_{tot}^{-1}$	3.27E+00	1.17E+00	35.8
ΔH_1	$J\ mol^{-1}$	-1.01E+05	6.16E+00	0.0
ΔH_2	$J\ mol^{-1}$	-7.61E+02	4.85E+02	-63.7
ΔH_3	$J\ mol^{-1}$	-2.87E+02	2.61E+02	-90.8
ΔH_7	$J\ mol^{-1}$	-2.60E+04	1.41E+03	-5.4
A_4	$mol_{C18:3}\ mol_{tot}^{-1}\ min^{-1}$	1.22E-01	7.89E-02	64.9
A_5	$mol_{C18:2}\ mol_{tot}^{-1}\ min^{-1}$	1.41E-02	1.64E-03	11.6
A_6	$mol_{C18:1}\ mol_{tot}^{-1}\ min^{-1}$	1.28E-02	1.63E-03	12.7
$E_{a,4}$	$J\ mol^{-1}$	5.73E+04	7.37E+01	0.1
$E_{a,5}$	$J\ mol^{-1}$	5.52E+04	1.71E+03	3.1
$E_{a,6}$	$J\ mol^{-1}$	6.43E+04	1.83E+03	2.8

WILEY-VCH

Table S.11. Covariance matrix of kinetic parameters regressed for Equation 11, i.e., for rate laws of HCSH of VO by Lindlar catalyst interpreted by the reaction mechanism in Scheme 1(d). Note: “E±XX” = “$\cdot 10^{\pm XX}$”

	B_1	B_2	B_3	B_7	ΔH_1	ΔH_2	ΔH_3	ΔH_7	A_4	A_5	A_6	$E_{a,4}$	$E_{a,5}$	$E_{a,6}$
B_1	7.51E-05	-1.33E-03	-4.83E-02	-7.37E-03	-1.81E-02	1.01E+00	-7.54E-01	-2.48E+00	-1.88E-04	-1.08E-05	8.12E-06	1.21E-01	2.95E+00	-3.26E+00
B_2	-1.33E-03	4.24E+01	5.48E+00	1.22E+00	3.34E+00	-1.46E+03	7.57E+02	-1.02E+03	-4.30E-01	2.69E-03	-1.71E-03	6.29E+01	1.31E+03	-1.27E+03
B_3	-4.83E-02	5.48E+00	3.14E+02	1.28E+01	-5.01E+01	-8.37E+02	2.36E+03	-1.46E+04	4.10E-01	-1.48E-04	-1.31E-02	7.69E+02	1.79E+04	-1.89E+04
B_7	-7.37E-03	1.22E+00	1.28E+01	1.37E+00	-5.56E-01	-1.77E+02	1.91E+02	-4.66E+02	2.37E-02	1.31E-03	-1.80E-03	2.57E+01	5.78E+02	-5.99E+02
ΔH_1	-1.81E-02	3.34E+00	-5.01E+01	-5.56E-01	3.80E+01	-8.28E+02	-1.46E+01	7.97E+03	-8.14E-02	4.02E-03	4.42E-04	-4.10E+02	-9.66E+03	1.04E+04
ΔH_2	1.01E+00	-1.46E+03	-8.37E+02	-1.77E+02	-8.28E+02	2.35E+05	-9.51E+04	6.42E+04	1.15E+01	-2.81E-01	2.61E-01	-4.88E+03	-8.82E+04	7.51E+04
ΔH_3	-7.54E-01	7.57E+02	2.36E+03	1.91E+02	-1.46E+01	-9.51E+04	6.81E+04	-1.42E+05	-2.47E+00	1.59E-01	-2.50E-01	8.09E+03	1.78E+05	-1.81E+05
ΔH_7	-2.48E+00	-1.02E+03	-1.46E+04	-4.66E+02	7.97E+03	6.42E+04	-1.42E+05	1.98E+06	-8.73E+00	5.33E-01	5.61E-01	-1.03E+05	-2.41E+06	2.57E+06
A_4	-1.88E-04	-4.30E-01	4.10E-01	2.37E-02	-8.14E-02	1.15E+01	-2.47E+00	-8.73E+00	6.22E-03	-1.64E-06	-2.67E-05	3.83E-01	1.02E+01	-1.17E+01
A_5	-1.08E-05	2.69E-03	-1.48E-04	1.31E-03	4.02E-03	-2.81E-01	1.59E-01	5.33E-01	-1.64E-06	2.68E-06	-1.95E-06	-2.57E-02	-6.34E-01	7.04E-01
A_6	8.12E-06	-1.71E-03	-1.31E-02	-1.80E-03	4.42E-04	2.61E-01	-2.50E-01	5.61E-01	-2.67E-05	-1.95E-06	2.65E-06	-3.13E-02	-6.99E-01	7.20E-01
$E_{a,4}$	1.21E-01	6.29E+01	7.69E+02	2.57E+01	-4.10E+02	-4.88E+03	8.09E+03	-1.03E+05	3.83E-01	-2.57E-02	-3.13E-02	5.43E+03	1.26E+05	-1.34E+05
$E_{a,5}$	2.95E+00	1.31E+03	1.79E+04	5.78E+02	-9.66E+03	-8.82E+04	1.78E+05	-2.41E+06	1.02E+01	-6.34E-01	-6.99E-01	1.26E+05	2.94E+06	-3.13E+06
$E_{a,6}$	-3.26E+00	-1.27E+03	-1.89E+04	-5.99E+02	1.04E+04	7.51E+04	-1.81E+05	2.57E+06	-1.17E+01	7.04E-01	7.20E-01	-1.34E+05	-3.13E+06	3.34E+06

WILEY-VCH

Table S.12. Kinetic parameters regressed for Equation 12, i.e., for rate laws of HCSH of VO by Lindlar catalyst interpreted by the reaction mechanism in Scheme 1(e). Note: "E±XX" = "$\cdot 10^{\pm XX}$"

Parameter	Units	*Value* x_n	SE_n	RSE_n (%)
B_1	bar_{H2}^{-1}	2.28E-01	6.70E-02	29.4
B_2	$mol_{tot}\ mol_{C18:3}^{-1}$	5.23E+00	4.89E+00	93.6
B_3	$mol_{tot}\ mol_{C18:2}^{-1}$	4.19E+01	1.11E+01	26.5
B_7	$mol_{C18:1}\ mol_{tot}^{-1}$	2.26E+00	1.07E+00	47.3
ΔH_1	J mol^{-1}	-1.28E+05	7.17E+03	-5.6
ΔH_2	J mol^{-1}	-4.11E+03	3.55E+03	-86.3
ΔH_3	J mol^{-1}	-6.91E+04	5.87E+03	-8.5
ΔH_7	J mol^{-1}	-1.25E+04	5.93E+03	-47.5
A_4	$mol_{C18:3}\ mol_{tot}^{-1}\ min^{-1}$	6.53E-01	6.84E-01	104.8
A_5	$mol_{C18:2}\ mol_{tot}^{-1}\ min^{-1}$	6.21E-02	9.82E-03	15.8
A_6	$mol_{C18:1}\ mol_{tot}^{-1}\ min^{-1}$	4.98E-02	1.37E-02	27.4
$E_{a,4}$	J mol^{-1}	5.62E+03	4.46E+03	79.3
$E_{a,5}$	J mol^{-1}	7.41E+04	6.13E+03	8.3
$E_{a,6}$	J mol^{-1}	1.61E+03	1.25E+03	77.8

WILEY-VCH

Table S.13. Covariance matrix of kinetic parameters regressed for Equation 12, i.e., for rate laws of HCSH of VO by Lindlar catalyst interpreted by the reaction mechanism in Scheme 1(e). Note: "E±XX" = "$\cdot 10^{\pm XX}$"

	B_1	B_2	B_3	B_7	ΔH_1	ΔH_2	ΔH_3	ΔH_7	A_4	A_5	A_6	$E_{a,4}$	$E_{a,5}$	$E_{a,6}$
B_1	4.49E-03	4.22E-03	4.30E-01	6.33E-02	1.57E+02	1.08E+02	-1.28E+02	-1.26E+02	4.89E-03	-2.01E-04	-8.48E-04	-2.65E+01	-1.51E+02	-1.48E+01
B_2	4.22E-03	2.39E+01	-1.73E+01	-4.37E-01	1.03E+04	-1.23E+04	3.49E+02	-5.64E+03	-3.10E+00	7.79E-03	-8.33E-03	-1.32E+04	3.14E+02	3.32E+03
B_3	4.30E-01	-1.73E+01	1.23E+02	8.51E+00	-4.73E+03	2.93E+04	-1.24E+04	9.90E+03	3.64E+00	-5.94E-03	-5.84E-02	2.27E+04	1.04E+04	-2.31E+03
B_7	6.33E-02	-4.37E-01	8.51E+00	1.15E+00	1.19E+03	2.06E+03	-2.30E+03	7.28E+02	2.08E-01	4.25E-04	-1.16E-02	9.68E+02	3.81E+02	-1.35E+02
ΔH_1	1.57E+02	1.03E+04	-4.73E+03	1.19E+03	5.14E+07	-3.59E+06	-3.33E+07	-2.00E+07	-1.83E+03	-4.25E+01	-5.84E+01	-1.94E+07	-7.97E+06	2.44E+05
ΔH_2	1.08E+02	-1.23E+04	2.93E+04	2.06E+03	-3.59E+06	1.26E+07	-4.33E+06	3.43E+06	1.95E+03	-5.32E+00	-1.35E+01	9.35E+06	-1.22E+05	-2.64E+06
ΔH_3	-1.28E+02	3.49E+02	-1.24E+04	-2.30E+03	-3.33E+07	-4.33E+06	3.44E+07	-2.56E+06	6.73E+00	2.06E+01	4.00E+01	1.76E+06	-2.74E+06	1.00E+06
ΔH_7	-1.26E+02	-5.64E+03	9.90E+03	7.28E+02	-2.00E+07	3.43E+06	-2.56E+06	3.52E+07	1.32E+03	4.31E+01	3.29E+01	1.83E+07	2.67E+07	2.27E+05
A_4	4.89E-03	-3.10E+00	3.64E+00	2.08E-01	-1.83E+03	1.95E+03	6.73E+00	1.32E+03	4.68E-01	5.14E-05	4.26E-04	2.09E+03	4.82E+02	-3.98E+02
A_5	-2.01E-04	7.79E-03	-5.94E-03	4.25E-04	-4.25E+01	-5.32E+00	2.06E+01	4.31E+01	5.14E-05	9.64E-05	4.84E-05	1.55E+01	3.80E+01	3.69E+00
A_6	-8.48E-04	-8.33E-03	-5.84E-02	-1.16E-02	-5.84E+01	-1.35E+01	4.00E+01	3.29E+01	4.26E-04	4.84E-05	1.86E-04	2.13E+01	2.77E+01	1.76E+00
$E_{a,4}$	-2.65E+01	-1.32E+04	2.27E+04	9.68E+02	-1.94E+07	9.35E+06	1.76E+06	1.83E+07	2.09E+03	1.55E+01	2.13E+01	1.99E+07	1.09E+07	-1.38E+06
$E_{a,5}$	-1.51E+02	3.14E+02	1.04E+04	3.81E+02	-7.97E+06	-1.22E+05	-2.74E+06	2.67E+07	4.82E+02	3.80E+01	2.77E+01	1.09E+07	3.76E+07	1.56E+06
$E_{a,6}$	-1.48E+01	3.32E+03	-2.31E+03	-1.35E+02	2.44E+05	-2.64E+06	1.00E+06	2.27E+05	-3.98E+02	3.69E+00	1.76E+00	-1.38E+06	1.56E+06	1.56E+06

WILEY-VCH

Table S.14. Kinetic parameters regressed for Equation 13, i.e., for rate laws of HCSH of VO by Lindlar catalyst interpreted by the reaction mechanism in Scheme 1(f). Note: "E±XX" = "·$10^{\pm XX}$"

Parameter	Units	***Value*** x_n	SE_n	RSE_n **(%)**
B_1	bar_{H2}^{-1}	4.14E-06	6.36E-06	153.6
B_2	bar_{H2}^{-1}	1.70E-02	7.40E-03	43.5
B_3	mol_{tot} $mol_{C18:3}^{-1}$	5.74E+00	8.30E+00	144.7
B_4	mol_{tot} $mol_{C18:2}^{-1}$	9.81E+00	4.06E+00	41.4
B_{11}	$mol_{C18:1}$ mol_{tot}^{-1}	6.84E-01	9.03E-02	13.2
ΔH_1	J mol^{-1}	-5.62E+04	1.21E-03	0.0
ΔH_2	J mol^{-1}	-4.15E+03	1.20E-02	0.0
ΔH_3	J mol^{-1}	-1.64E+04	3.61E-03	0.0
ΔH_4	J mol^{-1}	-1.56E+02	2.50E-01	-0.2
ΔH_{11}	J mol^{-1}	-4.44E+04	4.11E-03	0.0
A_5	$mol_{C18:3}$ mol_{tot}^{-1} min^{-1}	5.47E+01	3.64E-01	0.7
A_6	$mol_{C18:3}$ mol_{tot}^{-1} min^{-1}	1.41E-01	2.29E-01	161.6
A_7	$mol_{C18:2}$ mol_{tot}^{-1} min^{-1}	3.79E+00	5.59E+00	147.6
A_8	$mol_{C18:2}$ mol_{tot}^{-1} min^{-1}	1.85E-01	1.20E-01	64.9
A_9	$mol_{C18:1}$ mol_{tot}^{-1} min^{-1}	1.29E+00	8.28E-01	64.0
A_{10}	$mol_{C18:1}$ mol_{tot}^{-1} min^{-1}	1.31E-01	4.92E-02	37.5
$E_{a,5}$	J mol^{-1}	2.99E+03	2.07E-03	0.0
$E_{a,6}$	J mol^{-1}	1.05E+05	3.00E-05	0.0
$E_{a,7}$	J mol^{-1}	2.70E+05	1.72E-04	0.0
$E_{a,8}$	J mol^{-1}	7.71E+02	1.18E-01	0.0
$E_{a,9}$	J mol^{-1}	1.47E+05	3.39E-04	0.0
$E_{a,10}$	J mol^{-1}	3.51E+04	4.85E-03	0.0

WILEY-VCH

Table S.15. Covariance matrix of kinetic parameters regressed for Equation 13, i.e., for rate laws of HCSH of VO by Lindlar catalyst interpreted by the reaction mechanism in Scheme 1(f). Note: “E±XX” = “·10$^{±XX}$”

	B_1	B_2	B_3	B_4	B_{11}	ΔH_1	ΔH_2	ΔH_3	ΔH_4	ΔH_{11}	A_5	A_6	A_7	A_8	A_9	A_{10}	$E_{a,5}$	$E_{a,6}$	$E_{a,7}$	$E_{a,8}$	$E_{a,9}$	$E_{a,10}$
B_1	4.05E-11	2.83E-09	-4.79E-05	1.70E-05	5.76E-08	6.69E-09	-7.03E-08	-2.12E-08	-1.47E-06	-2.27E-08	1.57E-06	1.20E-06	-2.52E-05	-4.68E-07	9.21E-07	-1.87E-08	1.07E-08	-1.44E-10	5.13E-10	6.95E-07	1.99E-09	-2.75E-08
B_2	2.83E-09	5.48E-05	-1.57E-03	9.37E-03	2.12E-04	5.89E-07	-3.89E-06	-1.67E-06	-1.27E-04	-1.71E-06	5.75E-05	-3.26E-04	-9.38E-04	-6.20E-04	1.70E-03	-3.53E-04	2.08E-06	-1.19E-08	7.82E-08	5.29E-05	1.57E-07	-1.27E-06
B_3	-4.79E-05	-1.57E-03	6.88E+01	-2.38E+01	1.35E-02	-9.82E-03	9.58E-02	2.89E-02	2.01E+00	3.35E-02	-1.84E+00	-1.76E+00	2.99E+01	6.20E-01	-4.67E-01	-9.29E-04	-1.61E-02	1.74E-04	-9.16E-04	-9.50E-01	-2.73E-03	4.00E-02
B_4	1.70E-05	9.37E-03	-2.38E+01	1.65E+01	-3.75E-02	3.90E-03	-3.20E-02	-1.01E-02	-7.03E-01	-1.30E-02	4.08E-01	6.19E-01	-7.01E+00	-3.77E-01	-3.52E-01	-3.58E-02	7.04E-03	-4.53E-05	4.78E-04	3.31E-01	9.67E-04	-1.46E-02
B_{11}	5.76E-08	2.12E-04	1.35E-02	-3.75E-02	8.16E-03	-1.43E-05	-2.03E-04	-5.77E-05	-3.74E-03	5.40E-05	1.90E-02	-2.92E-03	-2.81E-01	5.54E-04	3.92E-02	-2.17E-03	-4.11E-05	-1.42E-06	-8.78E-06	1.80E-03	4.57E-06	3.58E-05
ΔH_1	6.69E-09	5.89E-07	-9.82E-03	3.90E-03	-1.43E-05	1.46E-06	-1.31E-05	-3.98E-06	-2.77E-04	-4.96E-06	2.05E-04	2.53E-04	-3.42E-03	-1.01E-04	-5.17E-05	2.59E-08	2.46E-06	-2.07E-08	1.59E-07	1.31E-04	3.79E-07	-5.81E-06
ΔH_2	-7.03E-08	-3.89E-06	9.58E-02	-3.20E-02	-2.03E-04	-1.31E-05	1.44E-04	4.33E-05	3.00E-03	4.44E-05	-3.50E-03	-2.43E-03	5.56E-02	7.77E-04	-2.42E-03	3.28E-05	-2.05E-05	3.13E-07	-8.60E-07	-1.42E-03	-4.06E-06	5.44E-05
ΔH_3	-2.12E-08	-1.67E-06	2.89E-02	-1.01E-02	-5.77E-05	-3.98E-06	4.33E-05	1.30E-05	9.01E-04	1.35E-05	-1.03E-03	-7.32E-04	1.64E-02	2.44E-04	-6.78E-04	1.16E-05	-6.28E-06	9.27E-08	-2.72E-07	-4.27E-04	-1.22E-06	1.64E-05
ΔH_4	-1.47E-06	-1.27E-04	2.01E+00	-7.03E-01	-3.74E-03	-2.77E-04	3.00E-03	9.01E-04	6.24E-02	9.38E-04	-7.06E-02	-5.07E-02	1.12E+00	1.72E-02	-4.84E-02	8.64E-04	-4.39E-04	6.36E-06	-1.94E-05	-2.96E-02	-8.46E-05	1.14E-03
ΔH_{11}	-2.27E-08	-1.71E-06	3.35E-02	-1.30E-02	5.40E-05	-4.96E-06	4.44E-05	1.35E-05	9.38E-04	1.69E-05	-6.80E-04	-8.62E-04	1.14E-02	3.41E-04	1.94E-04	-1.90E-06	-8.37E-06	6.90E-08	-5.48E-07	-4.44E-04	-1.29E-06	1.98E-05
A_5	1.57E-06	5.75E-05	-1.84E+00	4.08E-01	1.90E-02	2.05E-04	-3.50E-03	-1.03E-03	-7.06E-02	-6.80E-04	1.32E-01	4.48E-02	-2.03E+00	-6.87E-03	1.72E-01	-2.36E-03	2.50E-04	-1.08E-05	-1.32E-05	3.36E-02	9.40E-05	-9.44E-04
A_6	1.20E-06	-3.26E-04	-1.76E+00	6.19E-01	-2.92E-03	2.53E-04	-2.43E-03	-7.32E-04	-5.07E-02	-8.62E-04	4.48E-02	5.22E-02	-7.32E-01	-1.26E-02	-1.03E-02	2.60E-03	4.09E-04	-4.23E-06	2.44E-05	2.41E-02	6.94E-05	-1.03E-03
A_7	-2.52E-05	-9.38E-04	2.99E+01	-7.01E+00	-2.81E-01	-3.42E-03	5.56E-02	1.64E-02	1.12E+00	1.14E-02	-2.03E+00	-7.32E-01	3.12E+01	1.26E-01	-2.56E+00	3.51E-02	-4.34E-03	1.67E-04	1.59E-04	-5.35E-01	-1.50E-03	1.56E-02
A_8	-4.68E-07	-6.20E-04	6.20E-01	-3.77E-01	5.54E-04	-1.01E-04	7.77E-04	2.44E-04	1.72E-02	3.41E-04	-6.87E-03	-1.26E-02	1.26E-01	1.44E-02	-2.12E-03	3.47E-03	-1.89E-04	9.28E-07	-1.36E-05	-8.04E-03	-2.36E-05	3.81E-04
A_9	9.21E-07	1.70E-03	-4.67E-01	-3.52E-01	3.92E-02	-5.17E-05	-2.42E-03	-6.78E-04	-4.84E-02	1.94E-04	1.72E-01	-1.03E-02	-2.56E+00	-2.12E-03	6.85E-01	-1.66E-02	-1.17E-04	-1.38E-05	-6.71E-05	2.22E-02	5.80E-05	-2.32E-05
A_{10}	-1.87E-08	-3.53E-04	-9.29E-04	-3.58E-02	-2.17E-03	2.59E-08	3.28E-05	1.16E-05	8.64E-04	-1.90E-06	-2.36E-03	2.60E-03	3.51E-02	3.47E-03	-1.66E-02	2.42E-03	-4.42E-06	2.18E-07	7.90E-07	-3.69E-04	-1.00E-06	-2.38E-06
$E_{a,5}$	1.07E-08	2.08E-06	-1.61E-02	7.04E-03	-4.11E-05	2.46E-06	-2.05E-05	-6.28E-06	-4.39E-04	-8.37E-06	2.50E-04	4.09E-04	-4.34E-03	-1.89E-04	-1.17E-04	-4.42E-06	4.30E-06	-2.79E-08	3.01E-07	2.07E-04	6.03E-07	-9.65E-06
$E_{a,6}$	-1.44E-10	-1.19E-08	1.74E-04	-4.53E-05	-1.42E-06	-2.07E-08	3.13E-07	9.27E-08	6.36E-06	6.90E-08	-1.08E-05	-4.23E-06	1.67E-04	9.28E-07	-1.38E-05	2.18E-07	-2.79E-08	9.03E-10	4.70E-10	-3.02E-06	-8.50E-09	9.21E-08
$E_{a,7}$	5.13E-10	7.82E-08	-9.16E-04	4.78E-04	-8.78E-06	1.59E-07	-8.60E-07	-2.72E-07	-1.94E-05	-5.48E-07	-1.32E-05	2.44E-05	1.59E-04	-1.36E-05	-6.71E-05	7.90E-07	3.01E-07	4.70E-10	2.96E-08	9.09E-06	2.75E-08	-5.91E-07
$E_{a,8}$	6.95E-07	5.29E-05	-9.50E-01	3.31E-01	1.80E-03	1.31E-04	-1.42E-03	-4.27E-04	-2.96E-02	-4.44E-04	3.36E-02	2.41E-02	-5.35E-01	-8.04E-03	2.22E-02	-3.69E-04	2.07E-04	-3.02E-06	9.09E-06	1.40E-02	4.01E-05	-5.42E-04
$E_{a,9}$	1.99E-09	1.57E-07	-2.73E-03	9.67E-04	4.57E-06	3.79E-07	-4.06E-06	-1.22E-06	-8.46E-05	-1.29E-06	9.40E-05	6.94E-05	-1.50E-03	-2.36E-05	5.80E-05	-1.00E-06	6.03E-07	-8.50E-09	2.75E-08	4.01E-05	1.15E-07	-1.56E-06
$E_{a,10}$	-2.75E-08	-1.27E-06	4.00E-02	-1.46E-02	3.58E-05	-5.81E-06	5.44E-05	1.64E-05	1.14E-03	1.98E-05	-9.44E-04	-1.03E-03	1.56E-02	3.81E-04	-2.32E-05	-2.38E-06	-9.65E-06	9.21E-08	-5.91E-07	-5.42E-04	-1.56E-06	2.35E-05

WILEY-VCH

WILEY-VCH
